# From a Cochineal Dye to the Possibility of an Anticancer Agent: Free Radical‐Mediated Activity of Carminic Acid and Its Co^II^ Complex on Nucleobases, DNA, and PC3 Prostate Carcinoma Cells

**DOI:** 10.1002/cbdv.202501312

**Published:** 2026-10-21

**Authors:** Tusar Kanti Mandal, Argha Dasgupta, Tanmoy Saha, Sougata Ghosh Chowdhury, Parimal Karmakar, Saurabh Das

**Affiliations:** ^1^ Department of Chemistry (Inorganic Section) Jadavpur University Kolkata India; ^2^ Department of Life Science and Biotechnology Jadavpur University Kolkata India

**Keywords:** carminic acid, Co^II^‐carminic acid, DNA, glassy carbon electrode, nucleobase, semiquinone‐radical anion

## Abstract

Anthracycline‐based anticancer agents perform through different modes; the free radical pathway is just one of them. Although they are effective anticancer agents, for different reasons, there is a lookout for simpler analogs that mimic their actions. Carminic acid, having a close resemblance to anthracyclines, and its Co^II^‐complex were investigated for activity related to the generation of semiquinone. The compounds were reduced at their respective reduction potentials, which were previously identified using cyclic voltammetry. Interactions between reduced species of the compounds and biological targets (nucleobases or DNA) maintained in the immediate vicinity of the generation of reduced species were monitored, enabling a realization of what might happen when anthracyclines are reduced within cells. Changes caused to nucleobases were realized using HPLC, while those in DNA were followed by monitoring the decrease in fluorescence when the target DNA was subsequently treated with ethidium bromide. Results indicate that, in the presence of the Co^II^‐complex, the damage observed in uracil was approximately double, and that in cytosine and guanine was one and a half times greater than that observed when carminic acid was used as the sensitizer. For thymine and adenine, there was no significant difference in the results whether performed in the presence of carminic acid or its Co^II^‐complex. Studies on calf thymus DNA indicate modification caused in presence of the complex was one and a half times more than when carminic acid was used. Results indicate that the reduced species formed on the complex were more inclined to interact with cytosine and guanine than with thymine and adenine. Studies on PC3 (carcinoma) and WI‐38 (normal) cells reveal that the complex was more effective on carcinoma cells. On WI‐38, both compounds show relatively high IC_50_ values, suggesting they weren't much affected. Since DCFDA assay helps in realizing aspects related to ROS generation, increased ROS production provides an explanation for the reactivity of compounds on nucleobases, DNA, and cells. Hence, it may be said there is a strong possibility simpler anthracycline analogs like carminic acid and its Co^II^ complex could be effective as anticancer agents like the anthracyclines.

## Graphical abstract

Synthesis and characterization of a Co(II) complex of Carminic acid and free radical mediated damage by Carminic acid and its Co(II) complex on biological targets.

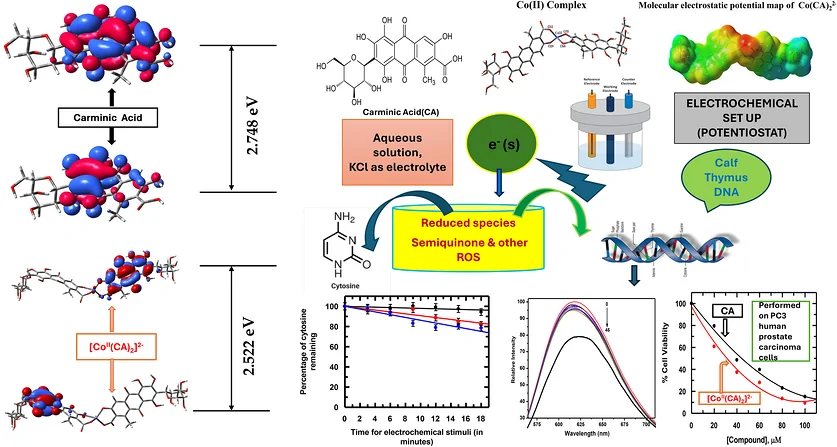

## 1 Introduction

Anthracyclines like doxorubicin and idarubicin are among the most discussed antibiotics in cancer chemotherapy used for the treatment of different malignancies [1, 2, 3, 4, 5, 6]. Their efficacy is, however, limited by adverse effects like irreversible cardiotoxicity and therapy‐related malignancy that are almost inseparable from aspects of drug action, thus requiring serious attention [5, 6, 7, 8, 9, 10]. Cardiotoxicity, an adverse effect of chemotherapy associated with this family of drugs, affects both short‐ and long‐term quality of life [6, 8, 9, 10, 11]. Hence, during administration of anthracyclines on patients, particularly children, several aspects are simultaneously taken into consideration [6, 7, 8, 12, 13, 14]. Patients on whom anthracyclines are administered are advised clinical monitoring often for their entire life [5, 6, 7, 8, 9, 10, 12, 13, 14]. Then, there are problems of drug resistance that are a hindrance to effective treatment [3].

Facts like those mentioned above make it apparent for an investigation into the mechanism of anthracycline activity and its imparting of toxic side effects that include a lot of therapy‐related stress [14, 15, 16, 17]. The aim of this work is to overcome the latter without having to sacrifice much on grounds of efficacy [2, 5, 6, 8, 14, 17]. It is well documented that doxorubicin, like many drugs, bring about double‐strand modification of DNA owing to its ability to intercalate and that it also poisons human DNA topoisomerase II [18, 19, 20, 21]. More recently, it was shown to cause chromatin damage, achieved through the eviction of histones from select sites in the genome [22]. Evaluation of similar such activities in several other anthracyclines and analogs reveals chromatin damage has a major contribution to efficacy. This pathway, however, does not associate itself with cardiotoxicity [22, 23, 24]. In a good number of cases, it is seen that in trying to arrive at a compound that would be efficient but less costly and have less toxic side effects, a compromise is made with efficacy. Since the primary intention of any drug action should be its efficacy, which should never be compromised, hence in trying to decrease toxic side effects by modifying anthracyclines, if a compromise is made this should be looked into [2, 3, 5, 6, 7, 8, 12, 13, 14, 15, 16, 25]. Another approach has been to select appropriate structural analogs, much simpler than anthracyclines but those that resemble them effectively [23, 26, 27, 28, 29, 30, 31]. However, in both approaches there is an element of compromise that cannot be avoided. Therefore, to realize the full potential of anticancer agents, knowledge on different pathways associated with drug action is essential [32, 33, 34, 35, 36]. One such mechanism for anthracyclines is the formation of reduced species where a quinone moiety participates in reversible electron transfer, generating either semiquinone or quinone dianion that is in turn capable of interaction with biological targets either in the reduced state itself or as other chemical species generated within cells [2, 23, 30, 31, 32, 33]. Any biological transformation occurring as a consequence of such interaction within cells is termed drug action. Another attribute of anthracyclines is their ability to interact with DNA [2, 30, 32, 33, 34, 35]. Hence, an attempt to design anticancer agents that mimic anthracyclines must have a structural similarity [19, 20, 30, 32, 33, 34, 35, 36]. Carminic acid is an important choice in this regard [37]. Besides being an anthraquinone, it has a sugar moiety in its structure, but the point of attachment of sugar in carminic acid is different from that in anthracyclines. Owing to the presence of sugar, it probably acquires the importance of having such units in anthracyclines [38]. Carminic acid resembles anthracyclines more closely than hydroxy‐9,10‐anthraquinones not having sugar moieties [26, 27, 28, 30, 32, 33, 34, 35, 36]. Sugars are reported as ideal ligands in obtaining prodrugs targeted at receptors overexpressed in cancer cells [38]. In fact, several drawbacks were identified for hydroxy‐9,10‐anthraquinones considered simple analogs of anthracyclines [32, 33, 34, 35, 36]. In vitro studies show carminic acid has activity that is intermediate between that of anthracyclines and hydroxy‐9,10‐anthraquinones (not having a sugar moiety) [20].

Metal ion complexes of hydroxy‐9,10‐anthraquinones have been prepared and tried for biological activity [32, 33, 34, 35, 36, 39, 40]. A Cu^II^ complex of carminic acid was found to be more effective on ALL MOLT‐4 cells than carminic acid itself [20]. The complex was identified as an inhibitor of human DNA topoisomerase I and human DNA topoisomerase II [20]. It is often believed that metal ion complexes of anthracyclines or their analogs compromise with activity in the free radical pathway. Since several studies claim metal complexes of anthracyclines or their analogs decrease the formation of semiquinone (having an established role in drug action) and that it might affect efficacy, a proper investigation becomes necessary. Evidence suggests in vivo formation of semiquinone occurs either due to one‐electron reduction of a quinone or through comproportionation of a quinone and quinone‐dianion [2, 41, 42, 43]. Generation of such reactive intermediates initiates damage on DNA, highlighting their importance in a radical generation‐based pathway that could lead to cell cycle arrest or cell death [44, 45, 46, 47]. Other forms of reactive oxygen species (ROS) that might be generated also affect the DNA of cancer patients either during the progress of the disease or during treatment, making understanding of the radical‐based pathway important [2, 26, 27, 28, 29, 32, 33, 34, 35, 36].

While, on the one hand, metal complex formation decreases the generation of semiquinones, on the other hand, the presence of a redox‐active metal ion could modulate electron transfer, leading to greater generation of ROS that would not happen for an uncomplexed drug molecule [2, 28, 33, 40, 44]. Hence, an attempt was made to realize the contribution of a free radical pathway in causing damage to cellular material using carminic acid and its Co^II^ complex. Since Co^II^ is redox active, having an ability for potential synergistic reactive oxygen species (ROS) generation (via Co^II^/Co^III^), cycling is an efficient and sustainable approach in advanced oxidation processes. The synergy arises from continuous regeneration of Co^II^ from Co^III^ preventing catalyst (in biological medium, enzyme) deactivation, minimizing metal leaching, and yet accelerating production of radicals like •OH and non‐radicals like ^1^O_2_ [44, 45, 46, 47, 48]. This could be an advantage for the complex, but whether at all Co^II^ is involved in cell damage in pathways mentioned above requires to be seen. If yes, then its role in generating ROS might add to the versatility of the free radical pathway [33, 47, 48, 49]. To mimic what happens following an interaction of our compounds with electrons of the electron transport chain (in mitochondria), compounds were subjected to electrochemical reduction at their respective reduction potential using a potentiostat. Reduced species were then allowed to interact with biological targets maintained in the immediate vicinity of such generation [44, 45, 46, 47]. From damage caused to biological targets, aspects related to the free radical pathway could be realized. However, it should also be kept in mind that associated with the metal ion could be intrinsic cobalt ion toxicity, redox cycling, non‐specific ROS generation, and aspects like bioaccumulation [50, 51]. Hence, it would be essential to make sure that Co^II^ remains intact in the complex and there is minimum leaching so that it improves critical balance and pharmacological relevance (Figure 1).

**FIGURE 1:**
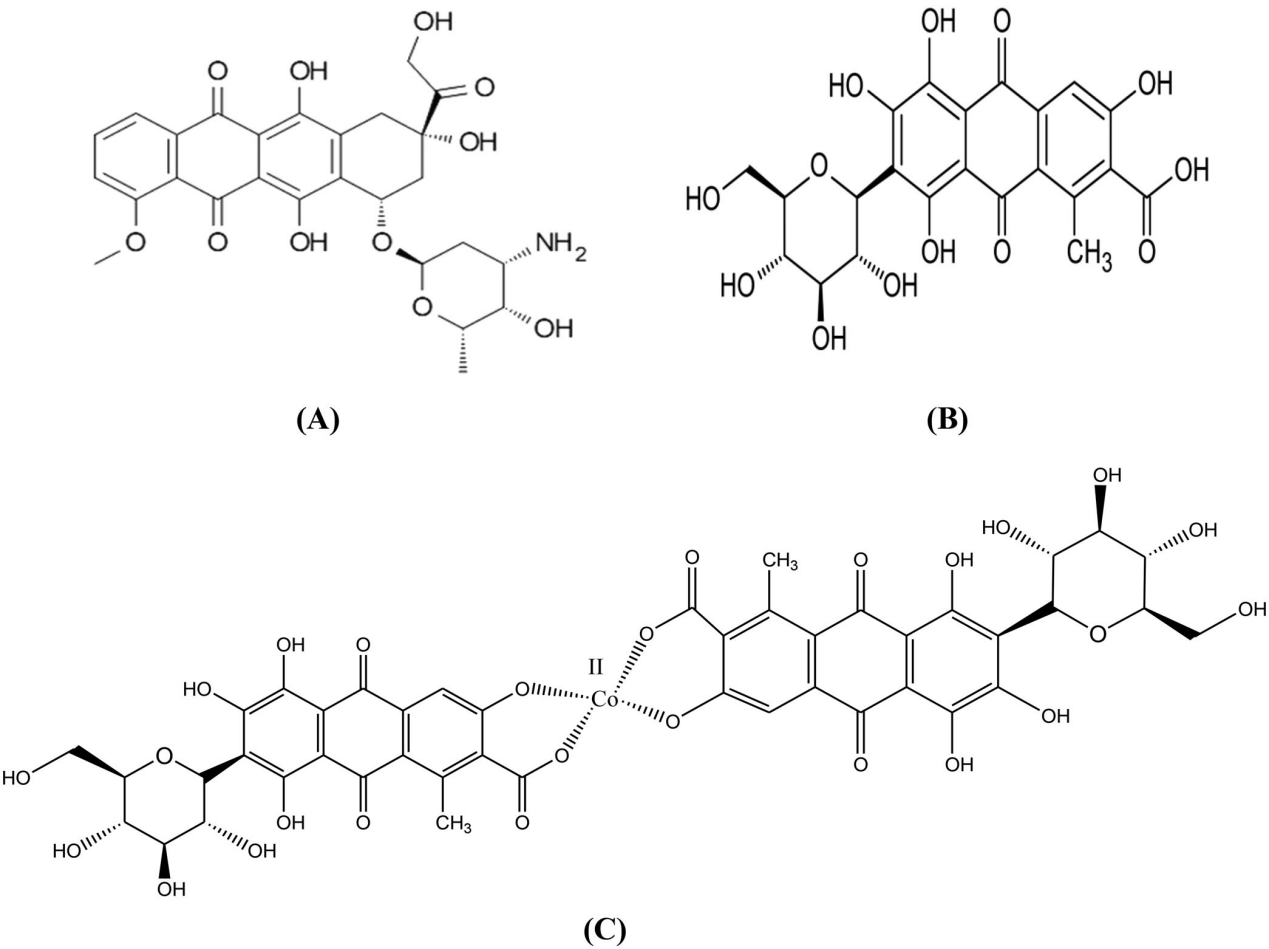
Representative structures of (A) doxorubicin, a standard anthracycline; (B) carminic acid, analog of anthracyclines; and (C) a Co^II^ complex of carminic acid.

## 2 Experimental

### 2.1 Materials

Carminic acid was purchased from Sigma Aldrich and purified by recrystallization. CoCl_2_ 6H_2_O and KCl (AR) were purchased from Merck India. KCl was used as an electrolyte for electrochemical experiments in aqueous solution. DPPH used for radical scavenging studies was purchased from SRL, India. Nucleobases (uracil, thymine, guanine, and adenine) and calf thymus DNA were procured from Sisco Research Laboratories, India. Cytosine was procured from Sigma Chemical Company. Ethidium bromide (EtBr) (CAS No. 1239‐45‐8) was procured from Merck, India. Sodium dihydrogen phosphate (AR) and disodium hydrogen phosphate (AR) used to prepare buffer solutions (pH ∼ 7.4) were procured from Merck, Germany. Triple distilled water was used for preparing solutions.

### 2.2 Instruments Used

pH was measured using a pH meter [Equip‐Tronics EQ‐610, India]. UV‐vis spectroscopy was performed on UV‐1700 Pharmasec, UV‐vis spectrophotometer, Shimadzu, Japan. Infrared spectroscopy was carried out using Spectrum Two, FTIR, spectrophotometer, Perkin Elmer. Fluorescence measurements were made on a Duetta Fluorescence and Absorbance Spectrometer, Horiba, Japan. Elemental analysis was performed using a Perkin‐Elmer 2400 II elemental analyzer. Mass spectra were recorded on aMicromass Q‐Tof micro TM (Waters Corporation) operating in ESI positive mode. DFT and molecular docking simulation were performed on a Linux operating system supported by a GPU‐NVIDIA RTX 3060 Ti and an Intel i7 9700kF processor. Cyclic voltammetry was performed using a Metrohm Autolab PGSTAT101 Potentiostat/Galvanostat electrochemical analyzer with a conventional three‐electrode system. The working electrode (glassy carbon) had a surface area of 0.0314 cm^2^, the counter electrode was a platinum wire, and the reference electrode was Ag/AgCl, satd. KCl. Before any experiment, the solution was degassed for nearly 30 min using high purity Ar. Any form of analysis related to cyclic voltammetry was done using NOVA 1.10.1.9 software. HPLC was performed on a Shimadzu Corporation, Japan, instrument. Analysis related to HPLC was done using LC Solutions software, Shimadzu. A C_18_ column (Phenomenex) was used as stationary phase and 5% methanol—95% water was the mobile phase; flow rate was 1 mL per min. Each nucleic acid base was followed at 254 nm. DPPH radical scavenging activity was followed using EPR spectroscopy. Measurements were made on a Jeol JES‐FA 200 ESR spectrometer equipped with Jeol microwave bridge. Spectroscopic parameters were 9.44 GHz (frequency), 100 mT (field sweep), 0.998 mW (microwave power), and a modulation amplitude of 3000 mT. EPR of samples was recorded using Jeol Quartz pyrex EPR tubes (no. 193 5D). Experimental data were fitted to equations using GraFit 3.0 and Origin 8.5.

### 2.3 Methods

#### 2.3.1 Stoichiometry of Complex Formation (Co^II^ With CA) by Mole Ratio and Job's Method

Stoichiometry of complex formation was ascertained by mixing different volumes of equimolar concentrations (100 µM) of Co^II^ and CA. In the mole ratio method, for one set of experiments, CA was kept constant and Co^II^ was varied, while in another, Co^II^ was constant and CA was varied. Absorbance at 510 nm was plotted against (T_M_/T_L_)_TL_ for the former and (T_L_/T_M_)_TM_ for the latter; T_M_ and T_L_ are concentrations for Co^II^ and CA, respectively. In Job's method of continuous variation, complementary mixtures were prepared using equimolar concentrations (100 µM) of stock solutions of Co^II^ and CA. Absorbance was recorded at 500 nm.

#### 2.3.2 Determination of pK_a_ of CA in Absence and Presence of Co^II^

Proton dissociation constants for CA were determined in aqueous solution and compared with values reported earlier [20]. The pK_a_ of CA in the presence of Co^II^ was determined. For CA, when present alone, absorbance was plotted against pH at 568 nm, while that in the presence of Co^II^ was followed at 557 nm. In solution, CA exists in four distinctly different forms, a molecular form: a mono‐anionic form (following removal of a proton on carboxyl), and di‐ or tri‐anionic forms where one or more phenolic –OH protons dissociate depending on the pH of the medium. Ionic strength was maintained at 0.01 M using NaNO_3_. Equations (1)–(3) explain dissociation of CA both in the absence and presence of Co^II^:

(1) $$\;QH_{2}H^{*}⇌QH^{--}+{H^{*}}^{+}\;K_{1}=\frac{\left[{H^{*}}^{+}\right]\left[QH^{--}\right]}{\left[\mathrm{QH}H^{*}\right]}$$

(2) $$\;Q{H_{2}}^{--}⇌QH^{2-}+H^{+}\;K_{2}=\frac{\left[QH^{2-}\right]\left[H^{+}\right]}{\left[Q{H_{2}}^{--}\right]}$$

(3) $$\;QH^{--}\;⇌\;Q^{3-}+H^{+}\;\;K_{3}=\frac{\left[Q^{3-}\right]\left[H^{+}\right]}{\left[QH^{--}\right]}$$

Absorbance was fitted to Equation (4), yielding pK_a1_, pK_a2_, and pK_a3_, respectively [20, 32, 33, 34, 35, 36]:

(4) $$\begin{array}{ccc} A_{\mathrm{obs}} & = & \frac{A_{1}}{\left(1+10^{\mathrm{pH}\;-\;pK_{1}}+10^{\mathrm{pH}\;-\;pK_{2}}+10^{\mathrm{pH}\;-\;pK_{3}}\right)}\;\;\\ & & +\frac{A_{2}}{\left(1+10^{pK_{1\;}-\;\mathrm{pH}}+10^{\mathrm{pH}\;-\;pK_{2}}+10^{\mathrm{pH}\;-\;pK_{3}}\right)}\\ & & +\frac{A_{3}}{\left(1+10^{pK_{1}\;-\;\mathrm{pH}}+10^{pK_{2\;}-\;\mathrm{pH}}+10^{\mathrm{pH}\;-\;pK_{3}}\right)}\\ & & +\frac{A_{4}}{\left(1+10^{pK_{1}\;-\;\mathrm{pH}}+10^{pK_{2\;}-\;\mathrm{pH}}+10^{pK_{3\;}-\;\mathrm{pH}}\right)} \end{array}$$

A_1_, A_2_, A_3_, and A_4_ are absorbance due to molecular, mono‐anionic, di‐ and tri‐anionic forms of CA, respectively. pK_a1_, pK_a2_, and pK_a3_ are the three pK_a_ values for dissociation of protons depicted by Equations (1)–(3). Spectrophotometric titrations were performed in the pH range 3.1 to 11.0 (Figure S1).

#### 2.3.3 Speciation Modeling

Aqueous speciation of Co^II^–carminic acid was modeled using the HySS 2009 program [52, 53]. The model incorporates the stepwise protonation equilibria of carminic acid and the stability constant of a Co^II^–carminate complex determined in this work, together with the hydrolysis equilibria of Co^II^ (CoOH^+^, Co(OH)_2_, Co(OH)_3_^−^, and Co_4_(OH)_4_^4^^+^) taken from the literature [54], and a check for the precipitation of Co(OH)_2_(s). All formation constants are referenced to the fully deprotonated ligand and used at an ionic strength of *I* = 0.01 M; as HySS 2009 program does not apply ionic‐strength corrections internally, constants were expressed at working ionic strength prior to input. Species distribution diagrams were computed as a function of pH (3.0–10.0) at total metal and ligand concentrations corresponding to each experiment. To verify calculations, the same equilibrium system was solved independently using a Newton–Raphson mass‐balance solver implemented in Python; both methods gave concordant results, and both input files may be provided on demand. Stability constants used were derived as described previously [55].

#### 2.3.4 Preparation of Co^II^(CA)_2_

An aqueous solution (20 mL) containing 0.1 mmol CoCl_2_.6H_2_O was gradually added under stirring conditions to a 20 mL solution of CA in ethanol containing 0.2 mmol. The mixture was transferred to a 100 mL round‐bottom flask, and the pH was adjusted to 6.3. It was warmed under reflux for 4 h. A reddish‐brown compound was formed which was filtered and recrystallized from an alcohol‐water mixture. Microanalytical data for Co^II^(CA)_2_^2−^ (= C_44_H_36_O_26_Co, FW = 1039.69) in %: C, 50.80; H, 3.49. Found: C, 50.32, H, 3.45. IR: ν(O–H) 3449 cm^−1^, ν(C–H) 2878–2940 cm^−1^, ν(C–O) 1057 cm^−1^. ESI‐MS spectrum (m/z): 1041.38. *λ*_max_ = 516 nm in DMSO.

#### 2.3.5 Computational Methodology

Computational analysis of the prepared compound and carminic acid was carried out on the basis of structures optimized in the ground state both for the gas phase and DMSO through polarizable continuum model (PCM) using integral equation formalism variant (IEFPCM), a default self‐consistent reaction field (SCRF) method. All quantum‐chemical calculations were performed using Gaussian 16 [56]. The system being open‐shell (having an odd number of electrons) unrestricted B3LYP level of theory (UB3LYP) with standard 6‐31G+(d,p) basis set for C, H, and O atoms in conjunction with LanL2DZ effective core potential basis set for Co was used [56, 57, 58] Ground state (*S*_0_) geometry in gaseous medium was fully optimized using the program's strict convergence criteria. Frequencies were determined on optimized geometries where all vibrational frequencies were found to be positive to ensure stationary points' global minima. The GaussView software was used to visualize frontier MOs [59, 60].

#### 2.3.6 Electrochemical Measurements

Electrochemical experiments on carminic acid and its Co^II^ complex were carried out in an air‐tight electrochemical cell (50 mL). Before each electrochemical experiment, the solution was degassed for ∼30 min using high purity argon. For each compound, reduction in aqueous solution was analyzed using the Randles‐Sevcik equation (Equation 5, Figure 2) to ensure that the process was diffusion‐controlled, an essential criterion for the experiments to follow [61]:

(5) $$I_{pc}=\left(2.69\cdot 10^{5}\right)n^{3/2}D_{0}^{1/2}AC_{n}^{1/2}$$

**FIGURE 2:**
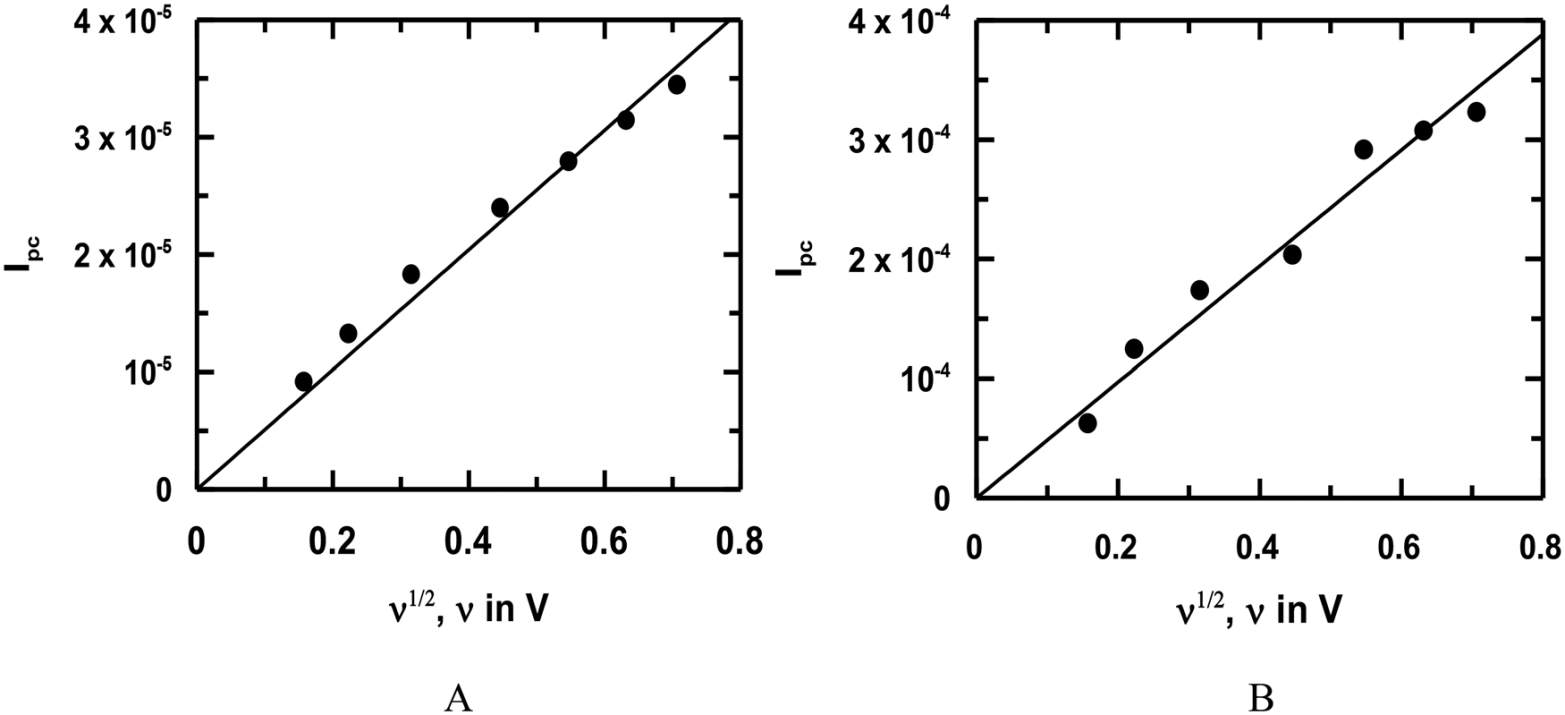
Plot of cathodic peak current (*I*_pc_) versus square root of scan rate (*ν*) for one‐electron reduction of (A) CA in aqueous solution at pH ∼ 7.4 and (B) Co^II^‐CA in ethanol‐DMSO (20% DMSO) solution at a potential of −0.65 and −0.70 V, respectively.

*I*_pc_ refers to current in amperes at cathodic peak potential, *n* is the total number of electrons, *D*_0_ is the diffusion coefficient of species, *A* is the area of electrode (in cm^2^), *C* is the concentration of compounds in moles/cm^3^, and *ν* is the scan rate in Vs^−1^. Figure 3A is a voltammogram of Carminic acid in aqueous solution from where its reduction potential was obtained (−0.65 V). In ethanol‐DMSO (20% DMSO) solution, reduction potential of carminic acid in the complex was found at −0.70 V (Figure 3B).

**FIGURE 3:**
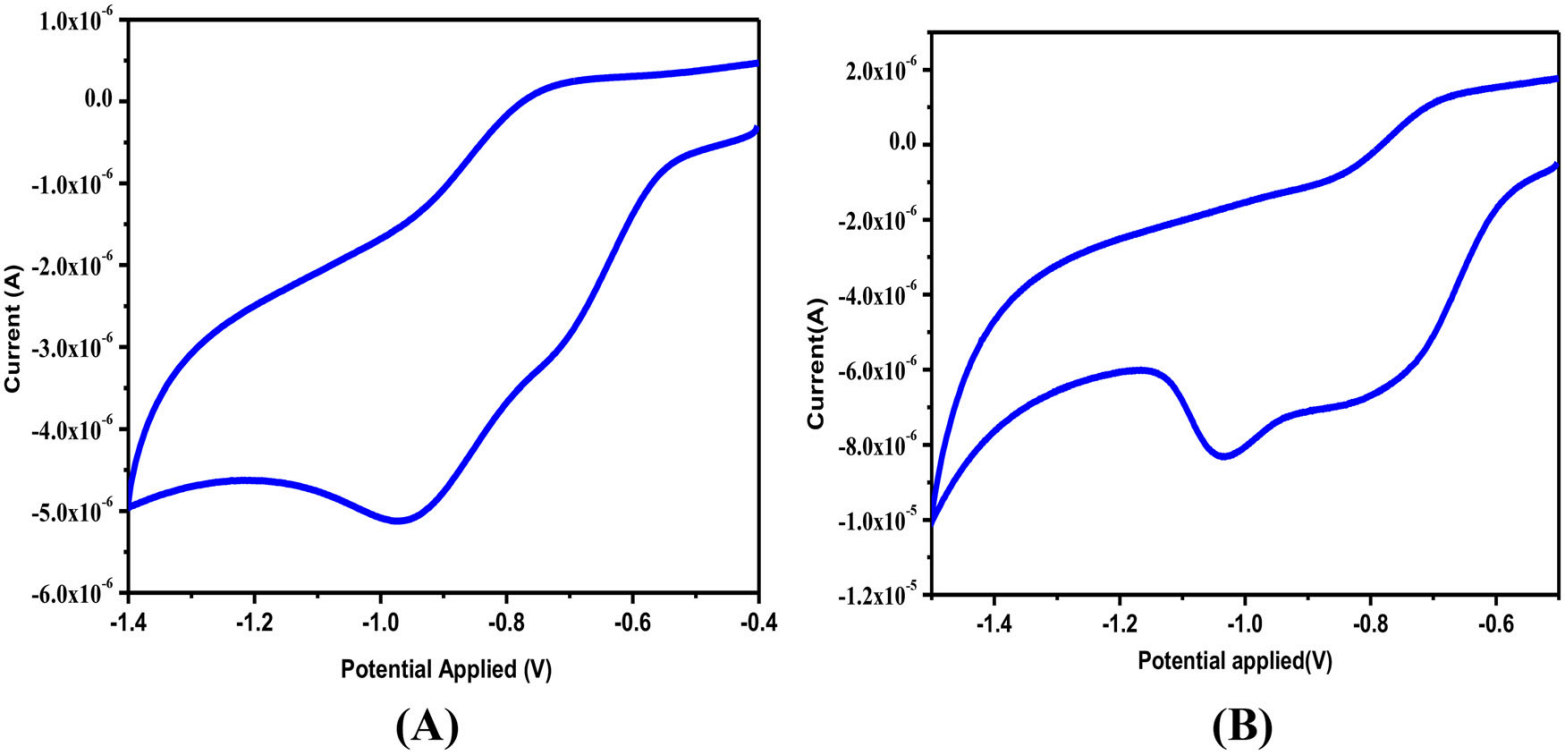
Cyclic voltammogram of (A) 1 mM carminic acid at pH ∼7.4 showing a single‐step‐two‐electron reduction of the quinone in aqueous solution containing 0.12 M KCl as electrolyte and (B) 1 mM of Co^II^ complex showing two successive one‐electron‐reduction of the quinone in ethanol‐DMSO (20% DMSO) having 0.12 M tetrabutyl ammonium bromide as electrolyte. A glassy carbon electrode of surface area 0.0314 cm^2^ was used; SCAN rate = 100 mV/sec.

Making use of reduction potentials of the compounds and using the same glassy carbon electrode, each compound was reduced for a specific time under deaerated (Ar saturated) conditions at constant pH (∼7.4) and constant potential (i.e., −0.65 V for CA and −0.70 V for Co^II^‐CA). While this was done, different nucleobases or double‐stranded calf thymus DNA were maintained in the immediate vicinity of reduced species formed on compounds.

With biological targets in the immediate vicinity of in situ electrochemically generated semiquinone, reduced species get an opportunity to interact with a target (either a nucleobase or DNA). Time for in situ electrochemical generation of species was constant so that a similar charge is provided to each compound during the experiment implying almost similar experimental error to be associated with the process (reduction of compounds using a potentiostat). Constancy of charge transferred onto compounds was verified by chrono‐amperometry enabling a comparison of results obtained following interaction of radical anions and/or radicals generated on compounds with target nucleobases (maintained in immediate vicinity of reduction) justifiable [44, 45]. Minor differences in chronoamperometry results could be due to differences in *D*_0_ values (diffusion coefficients of the species) for CA and Co^II^‐CA; however, the area (*A*) of the electrode (0.0314 cm^2^) was the same since the same electrode was used for CA and Co^II^‐CA. Semiquinone radical anion or protonated semiquinone under deaerated conditions interact with nucleobases or DNA in the electrochemical cell. For control experiments, target nucleobases or DNA were subjected to reduction at a potential intermediate between CA and its Co^II^ complex but with no compound (sensitizer) in solution, using the same glassy carbon electrode. All potentials were carefully applied since that formed the basis for experiments [44, 45]. The concentrations of nucleobases in solution were 10 times greater than those of compounds (sensitizers). For each experiment, aliquots were withdrawn at regular intervals of 3 min from the reaction vessel and subjected to HPLC for an analysis of damage caused to nucleobases.

#### 2.3.7 Analysis of Nucleobases Using High‐Performance Liquid Chromatography

Following providing electrochemical stimuli at different times, solutions having a nucleobase with or without additives were analyzed using HPLC. From HPLC chromatograms, the amount of each nucleobase remaining unaltered was obtained [44, 45, 62].

#### 2.3.8 Ethidium Bromide Fluorescence for Identification of Amount of DNA Not Modified

Any stimuli‐induced damage to DNA in the absence or presence of a sensitizer may be ascertained by treating aliquots with ethidium bromide (EtBr). To a fixed volume of aliquot withdrawn at regular intervals of 5 min, if a fixed volume of a solution of EtBr having a concentration at least thrice that of the concentration of DNA is added and fluorescence is recorded, then from the loss of fluorescence of the DNA‐EtBr adduct, it becomes possible to determine the amount of DNA modified [63, 64]. An adduct of DNA with EtBr, upon excitation at 510 nm, shows fluorescence emission at 602 nm. This was followed to know the amount of DNA remaining unaltered [43, 44, 63, 64]. In our case, loss of fluorescence of DNA‐EtBr adduct predicts the amount of calf thymus DNA having undergone modification following interaction either in a free radical pathway or due to physical interaction between the sensitizer and DNA [44, 45, 63, 64].

To understand how anthracyclines or their analogs interact with DNA through the free radical pathway, it was necessary to analyze the interactions between the sensitizer and nucleobases as well. In this study, this was done using a potentiostat as source of electrons [44, 45]. In situ reactivity of electrochemically generated semiquinone on carminic acid and its Co^II^ complex or other forms of ROS with calf thymus DNA, maintained in the immediate vicinity of their generation, helped to realize the role of semiquinone and/or superoxide radical anion on DNA, that is, whether damage was initiated through modification of nucleobases or was a consequence of physical interaction with DNA, such as binding or abstraction of hydrogen from a sugar unit. In this study, damage caused to nucleobases was followed. As identified earlier, complex formation of anthracyclines leads to a decrease in semiquinone generation [33, 34, 35, 36]. Hence, while the risk of cardiotoxicity might be under control [33, 34, 35, 36, 47], efficacy, particularly in the free radical pathway, could be affected.

However, we wish to mention here that this is a model study trying to mimic the aspect of electron procurement from an electron transport chain in mitochondria by reducing test compounds using a potentiostat. This was done to realize what happens when such compounds are reduced within cells in a “real life” drug action. It should also be mentioned here that the two are not the same (in fact completely different) and that ours is a model study trying to mimic things as close as possible within all forms of limitations.

#### 2.3.9 DPPH Radical Scavenging by EPR Spectroscopy

Electron paramagnetic resonance (EPR) measurements were performed at room temperature (298 K) to check for the stability of a freshly prepared solution of 2, 2‐diphenyl‐1‐picrylhydrazyl (DPPH) in methanol [65, 66, 67, 68]. Here DPPH denotes the stable radical DPPH•. This was done for 30 min. No significant loss in signal was detected. Previously, using a potentiostat, reduction of CA was carried out at −0.70 V in ethanol for one and a half hours. Thereafter, aliquot was withdrawn from that solution and immediately transferred to an ESR tube containing 6 mM, 500 µL DPPH solution in liquid N_2_ temperature. The solution was thoroughly mixed, and the EPR signal was recorded. Under exactly identical conditions, the EPR signal for DPPH alone was also recorded.

#### 2.3.10 Detection of Reactive Oxygen Species (ROS)

The fluorescent dye H_2_DCFDA (2',7'‐dichlorodihydrofluorescein diacetate) was used to identify the generation of ROS in living cells [69, 70, 71]. Cellular esterases deacetylate the dye after it enters cells, leading to a loss in fluorescence [69, 70, 71]. If ROS is present, a new compound (2',7'‐dichlorofluorescein) is formed, showing fluorescence at 520 nm (excitation at 485 nm) [69, 70, 71, 72]. Hence, if H_2_DCFDA is added to cells and fluorescence is still observed, it implies ROS is present in the cellular medium. Cells were seeded in 48‐well plates at a concentration of 6 × 10^4^ cells per well. For PC3 cell line, test compounds were added over a concentration range of 0 to 100 µM. Cells were incubated for 24 h. H_2_DCFDA (10 µM) was stained for 30 min in the dark at 37°C, 5% CO_2_, and 98% humidity. The study was performed in a manner where for one set of experiments no compounds were used, while for the other three sets IC_50_ concentration of CA, IC_50_ concentration of Co^II^ complex and a standard antioxidant N‐acetyl‐L‐cysteine (NAC) were used. For the antioxidant control, cells were pretreated with NAC (e.g., 5 mM, 1 h) prior to sample exposure, and ROS levels were quantified as mean fluorescence intensity normalized to vehicle. Fluorescence was determined (Biotek, USA) at 520 nm (emission) upon excitation at 485 nm.

#### 2.3.11 Cell Culture

WI‐38 and PC3 cells were cultured in DMEM media (Himedia) and RPMI‐1640 media (Himedia), respectively. Media were supplemented with 10% fetal bovine serum (Himedia), antibiotic mixture 1× penicillin–streptomycin solution (Himedia). Cells were incubated in a humidified CO_2_ incubator at 37°C.

#### 2.3.12 Cell Viability Assay

Both types of cells were seeded on a 96‐well plate at 5000 cells/well a day prior to the addition of compounds. Compounds were dissolved in DMSO (concentration < 0.5%). After 48 h incubation, cells were washed with 1× phosphate‐buffered saline and treated with MTT for 4 h at 37°C [19, 32, 34, 35, 36, 47]. Precipitates formed were dissolved in DMSO. The final DMSO concentration was maintained at ≤ 0.5% (v/v) in all conditions, and vehicle treatment alone did not affect cell viability. Plates were subsequently analyzed on a Biotek, USA, plate reader at 570 nm to determine IC_50_ (concentration essential for 50% cell killing). Experiments were performed in *n* = 3 independent biological replicates. Data are presented as mean ± SD. Statistical significance was assessed using one‐way ANOVA with Dunnett's post hoc test for multiple comparisons versus control. IC_50_ values were calculated by nonlinear regression using a four‐parameter logistic model.

## 3 Results and Discussion

### 3.1 Formation and Characterization of a Co^II^ Complex of Carminic Acid

#### 3.1.1 Determination of Stoichiometry

It was established through physicochemical experiments that complex formation leads to the generation of a 1:2 species, Co^II^(CA)_2_ (Figure S1A,B). Job's method of continuous variation (Figure S1C) also suggests the formation of a 1:2 Co^II^:CA species.

#### 3.1.2 Dissociation of Protons on CA in the Absence and Presence of Co^II^

Using Figure S2 and Equation (4), pK_a1_, pK_a2_, and pK_a3_ were determined for CA in solution (discussion in Supporting Information). Values were 5.43 ± 0.07, 8.53 ± 0.83, and 9.99 ± 0.70, respectively (Figure S3). Subsequently, CA and Co^II^ were mixed in the ratio determined through analysis on stoichiometry (Figure S3, SI). Solutions were titrated using NaOH.

Titration was followed spectrophotometrically over a pH range of 3.0 to 10.5 (Figure S4). It revealed that in the initial portion of the titration, a peak appeared at 492 nm in the pH range 3.10 to 4.73. At pH 5.30, it shifted slightly, showing a shoulder at 523 nm denoting dissociation of a proton from the carboxyl group in CA. The shoulder at 523 nm thereafter developed into a new peak at 526 nm and remained more or less constant at this wavelength for pH 5.30 to 8.20. Intensity at 526 nm increased as pH increased from 5.30 to 8.20, denoting dissociation of a phenolic‐OH proton on CA. Spectra at higher pH (8.92 to 10.0) underwent a further shift to higher wavelength, indicating the loss of a second pheolic –OH proton (Figure S4).

From Figure 4 and Equation (4), pK_a1_, pK_a2_, and pK_a3_ of CA in presence of Co^II^ were found to be 5.52 ± 0.03, 7.29 ± 0.62, and 9.61 ± 0.55, respectively (Figure 4). Stability constants *β* ^*^ and *β* were determined using Equations (S1–S5) [19, 20, 33, 34, 35, 36] and found to be 1.05 × 10^11^ (log *β* = 11.02).

**FIGURE 4:**
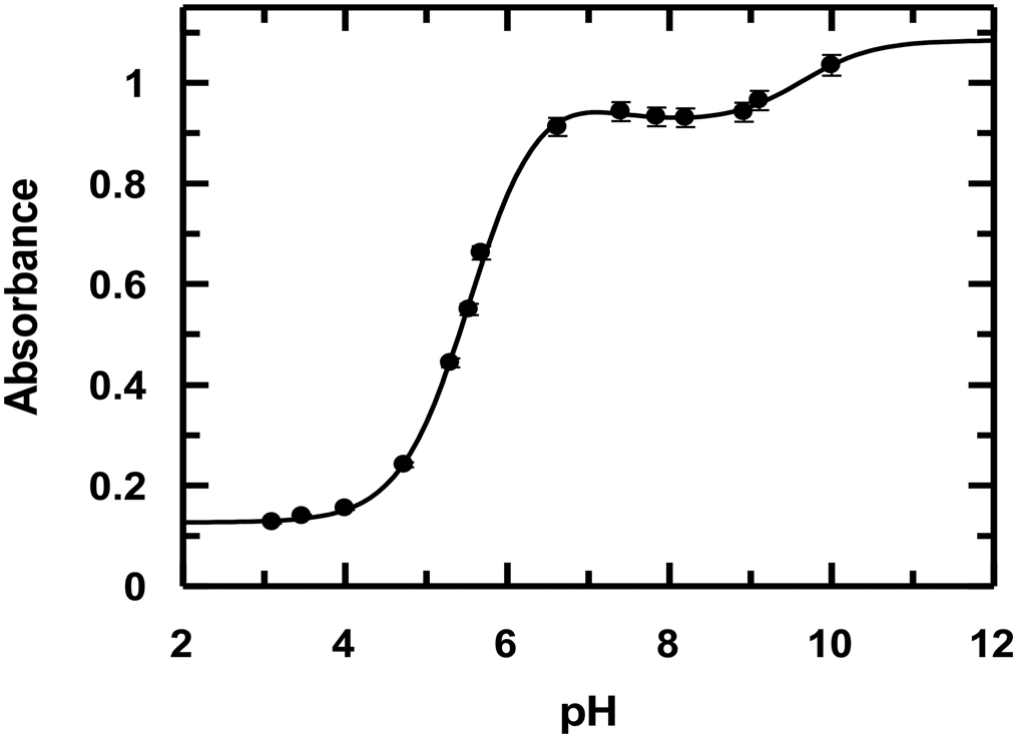
Spectrophotometric titration of CA in the presence of Co^II^ shown by a variation in absorbance at 557 nm; [CA] = 100 µM, [Co^II^] = 50 µM, [NaNO_3_] = 0.01 M, T = 301 K.

#### 3.1.3 FTIR Spectroscopy

Complex formation was ascertained by studying its IR spectrum and comparing it with CA (Figure S5). The IR spectrum of the complex has a broad band around 3392 cm^−1^ suggesting the presence of intramolecular hydrogen bonding between a carbonyl and neighboring phenolic –OH as observed in CA (Figure S5). The sharp band at 1353 cm^−1^ for CA got shifted to 1225 cm^−1^ in the complex. Peaks at 1577 cm^−1^ and 1040 cm^−1^ in CA were found at almost similar positions in the complex, suggesting quinone oxygen and phenolic‐OH adjacent to them are not involved in coordinating Co^II^. Complex formation occurs due to coordination of Co^II^ by carboxyl oxygen and its adjoining phenoxide oxygen.

#### 3.1.4 Mass Spectrometry

Considering Co^II^‐CA to be 1:2 species as realized from physico‐chemical experiments, a molecular ion peak was detected at 1041.3815 (m/z_theo_ = 1041.69) (Figure S6). From the molecular ion, loss of an –OH group could produce a fragment at m/z = 1024.38 (experimentally found at m/z = 1024.65). If from the molecular ion, the sugar unit along with four carbon atoms of the aromatic unit to which the sugar was attached departs from each ligand, a fragment having m/z = 519.38 is expected. Identification of a peak at m/z = 518.47 in the mass spectrum could be such a species. Hence, mass spectrum supports formation of a 1:2 Co^II^‐carminic acid complex.

#### 3.1.5 Magnetic Susceptibility

The compound was found to be paramagnetic (*μ* = 1.23 BM). However, the magnetic moment was much lower than that expected for one unpaired electron, suggesting the formation of a square planar complex [73, 74, 75]. The decreased value of the magnetic moment could be due to the interaction of two unpaired electrons, one on each Co^II^ present in a d_z_2 orbital and oriented perpendicular to the plane of coordination of the ligand toward the metal ion, leading to effective overlap. Overlap of two d_z_2 orbitals subsequently leads to partial spin pairing. Since it is not completely diamagnetic, this suggests the presence of an equilibrium between a spin‐paired state and an isolated spin‐free state for the compound.

#### 3.1.6 Conductance Measurements

Conductance measurements reveal complex shows maximum conductivity in water, followed by that in water‐DMSO (1:1), followed by that in pure DMSO, suggesting the presence of dianionic species in solution having the formula Na_2_[Co(CA)_2_]. Hence, from here on the compound is represented as [Co(CA)_2_]^2−^.

#### 3.1.7 Speciation Studies Concerning the Complex With Respect to Co^II^

To be able to realize the role of the complex in biological studies, it was essential we have some knowledge regarding its existence in solution. Since the identity of Co^II^ species present under experimental conditions should not be assumed from the standpoint of the stability constant alone, we carried out quantitative speciation analysis to realize the situation.

Using determined protonation and stability constants, we constructed an equilibrium speciation model in HySS 2009 program [52, 53] incorporating carminate protonation, formation of Co^II–^carminate complex, and Co^II^ hydrolysis species (CoOH^+^, Co(OH)_2_, Co(OH)_3_^−^, polynuclear [Co_4_ (OH)_4_^4^^+^] [54], together with a check for Co(OH)_2_(s) precipitation. All constants were used at an ionic strength of 0.01 M. The model was cross‐validated against an independent Newton–Raphson equilibrium solver written in Python. Both methods agreed within experimental uncertainty. The analysis clarifies the point that an intact fraction of the complex in solution is strongly dependent on concentration for this 1:2 metal–ligand system. At the concentration used to determine stability constant where [Co^II^] = 50 µM and [CA] = 100 µM, the complex accounts for ∼90% of total Co^II^ at pH 7.4, consistent with reported stability. However, at a lower (micromolar) concentration, that was used for electrochemical or “interaction with DNA” experiments where [Co(CA)_2_]^2−^ was 4.5 µM, the same constant predicts ∼57% of Co^II^ to be present as an intact complex at pH 7.4, with the remainder being labile as Co^II^ in other forms and above pH ∼8.5 as cobalt hydroxide species. Such speciation plots are provided in Supporting Information (Figure S7). Therefore, there would be the possibility that the complex is present in forms other than [Co(CA)_2_]^2−^ in different media.

There are two limitations to our analysis. First, this model describes aqueous complex formation as a function of pH and concentration; it does not incorporate competition from phosphate, carbonate, etc., present alongside it during experiments, where electrochemical stimuli were provided to compounds in presence of nucleobases and DNA or serum components present in cell‐culture media, nor does it account for Co^II^/Co^III^ redox inter‐conversion, which by its nature cannot represent a purely thermodynamic mass‐balance framework. Oxidation to Co^III^ is chemically feasible under prevailing conditions, and therefore we need to discuss such possibilities in the context of redox‐based mechanisms proposed for our H_2_DCFDA/ROS results and wherever else applicable; in case of electrochemical studies, rather than implying a single static species persist. Second, we retain coordination mode as assigned by FTIR and note unambiguous structural assignment of isolated complex has to be confirmed by additional methods.

A further limitation of this study is that stability and speciation of Co(CA)_2_^2−^ were not evaluated under physiological conditions pertaining to cell line studies. Since a Co^II^ complex could undergo hydrolysis, oxidation, or ligand exchange in biological media, it should not be said conclusively that an intact Co(CA)_2_^2−^ is the predominant intracellular species. In future studies with this compound, we would investigate its physicochemical stability in PBS and serum‐containing culture medium using time‐dependent spectroscopic and analytical approaches. Hence, it is mentioned here that species considered to be entering cells in our reported cell line studies cannot exclusively be [Co(CA)_2_]^2−^; but rather a pH‐ and concentration‐dependent equilibrium mixture. As already mentioned, we reiterate that there would be a possibility of the complex to be present in forms other than [Co(CA)_2_]^2−^ in different media. Hence, observed biological activity should best be interpreted as due to an intact complex along with labile fractions of Co^II^ depending on concentration and pH.

### 3.2 DFT Optimized Structure

The optimized shape of the Co^II^ complex of Carminic acid is shown in Figure 5. Figures 6 and 7 depict the molecular orbitals of the optimized molecule in DMSO at the spin‐α and spin‐β states. Table 1 provides a list of chosen bond lengths and bond angles. Geometry of the complex was found to be distorted square planar based on spectroscopic and magnetic susceptibility measurements [75, 76]. Following optimization, it was realized that energy of the molecule both in gas phase and DMSO was −103354 eV. Polarizability and dipole moment were 1323.83 a.u. and 38.38 Debye respectively; point group being C1.

**FIGURE 5:**
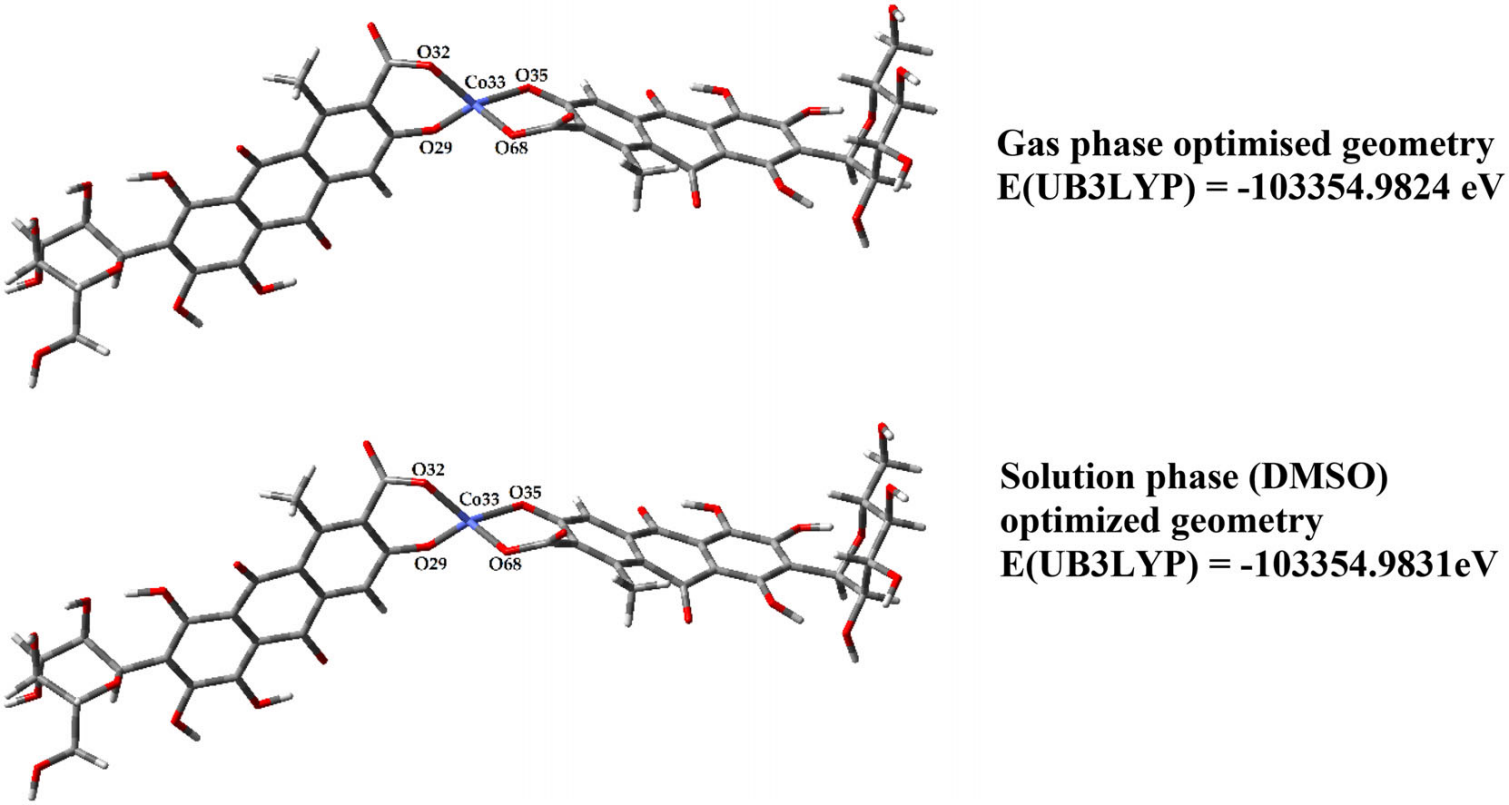
DFT‐optimized structure of Co^II^‐Carminic acid.

**FIGURE 6:**
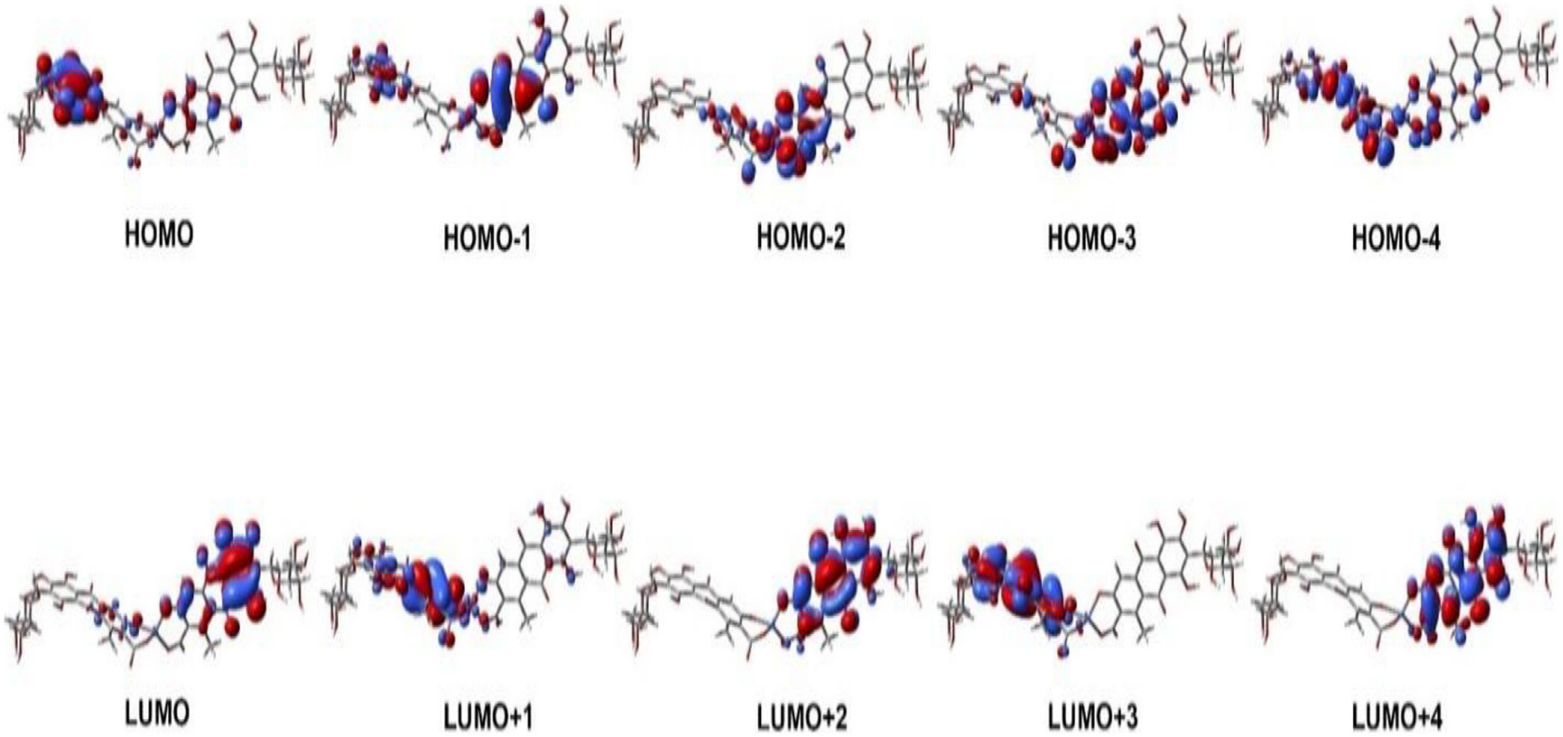
Frontier molecular orbitals (FMOs) of optimized geometry of Co^II^‐carminic acid complex in DMSO phase (α‐MOs).

**FIGURE 7:**
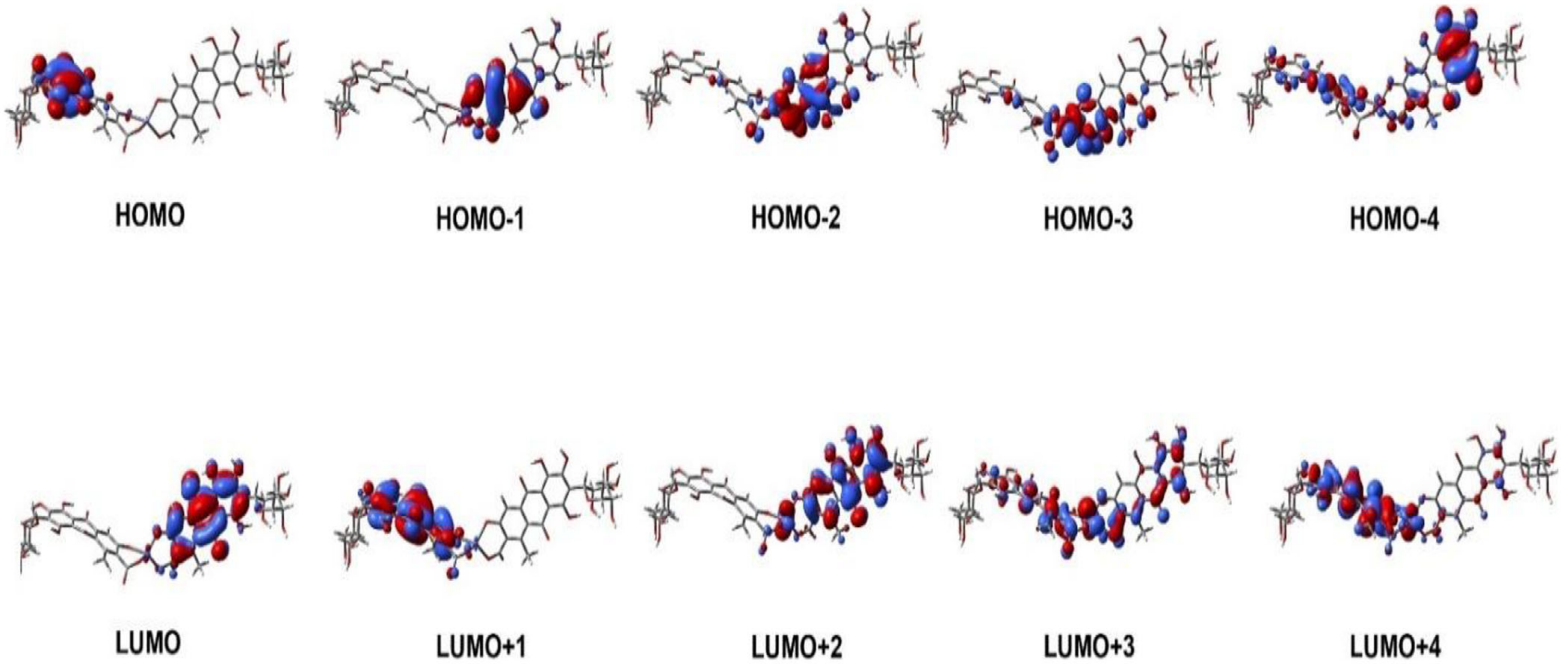
Frontier molecular orbitals (FMOs) of optimized geometry of Co^II^‐carminic acid complex in DMSO phase (β‐MOs).

**TABLE 1:** Coordinated bond distances (in Å) and bond angles (in °) of the Co^II^ complex.

Bond	Gaseous phase	DMSO phase	Angle	Gaseous phase	DMSO phase
Co33–O32	1.967	1.923	O32–Co33–O35	119.87	115.26
Co33–O35	1.948	1.942	O29–Co33–O68	106.41	105.43
Co33–O29	2.037	2.025	O29–Co33–O32	87.80	90.70
Co33–O68	1.951	1.948	O35–Co33–O68	91.50	91.17

These calculations describe the electronic structure of the proposed coordination model and do not constitute independent structural evidence; geometry being assumed from spectroscopic assignment rather than that determined computationally.

#### 3.2.1 A Frontier Molecular Orbital Analysis

Excitation characteristics and the capability for electron transport are predicted qualitatively using frontier molecular orbitals. Transition from ground state to first excited state (electronic absorption) is a movement of electrons from highest occupied molecular orbital (HOMO) to lowest unoccupied molecular orbital (LUMO). HOMO functions as electron donor and LUMO as electron acceptor. Chemical reactivity may be realized by energy levels of HOMO and LUMO and gaps associated with them. Table S1 represents HOMO and LUMO energies of carminic acid. Figure S8 shows HOMO to HOMO − 4 and LUMO to LUMO + 4 orbitals computed at B3LYP/6‐31 + G(d,p) level for carminic acid in DMSO. HOMO and LUMO energies were predicted as −6.271 and −3.523 eV respectively having an energy gap of 2.748 eV. Tables S2 and S3 reflect selected HOMO and LUMO energies of [Co(CA)_2_]^2−^ in spin α and spin β states. Figures 6 and 7 show the HOMO to HOMO − 4 and LUMO to LUMO + 4 orbitals computed at B3LYP/6‐31 + G(d,p) level for the complex in DMSO [43, 44]. For spin α state, HOMO and LUMO energies were predicted as −6.110 and −5.147 eV respectively with energy gap being 0.963 eV (Table S2). For the spin‐β state, the HOMO and LUMO energies were −6.165 and −3.643 eV, respectively, and the energy gap = 2.522 eV (Table S3). Figure 8 shows HOMO‐LUMO gap for spin α and spin β states.

**FIGURE 8:**
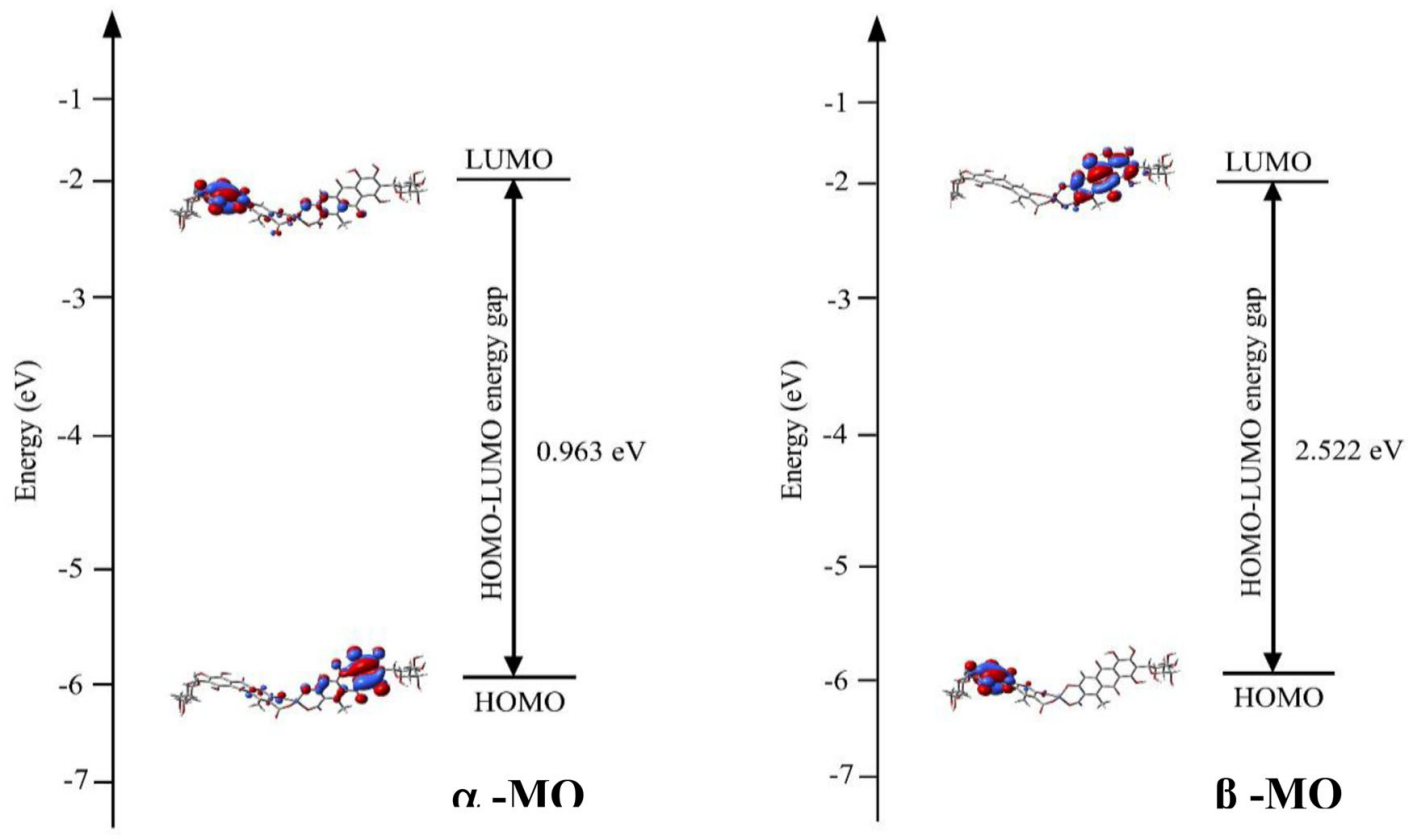
HOMO‐LUMO energy gap of Cobalt carminic acid complex in α and β MOs.

#### 3.2.2 Molecular Electrostatic Potential (MEP)

MEP is related to electronic density and serves as a useful descriptor for determining sites for electrophilic and nucleophilic reactions; suggestive of the possibility for hydrogen‐bonding. In case of Co(CA)_2_^2−^, MEP (Figure 9) depicts charge distribution on the molecule in three dimensions. Different values for electrostatic potential at the surface are depicted by different colors; red denotes regions with highest electronegative potential, blue is used for regions with highest positive potential and green for having zero potential. Potential regions change in the manner, red < orange < yellow < green < blue. Such maps are color‐coded from darkest red (−150.86 kJ/mol) to deepest blue (160.16 kJ/mol); blue denoting strong attraction and red strong repulsion. Lone pairs of electronegative atoms are typically linked to regions of negative potential. Such negative regions in Co(CA)_2_^2−^ are found located around oxygen of carbonyl groups, identified from MEP mapping (Figure 9). Maximum positive regions are located on hydrogens of O‐H that could be potential sites for nucleophilic attack having a maximum value of +160.16 kJ/mol.

**FIGURE 9:**
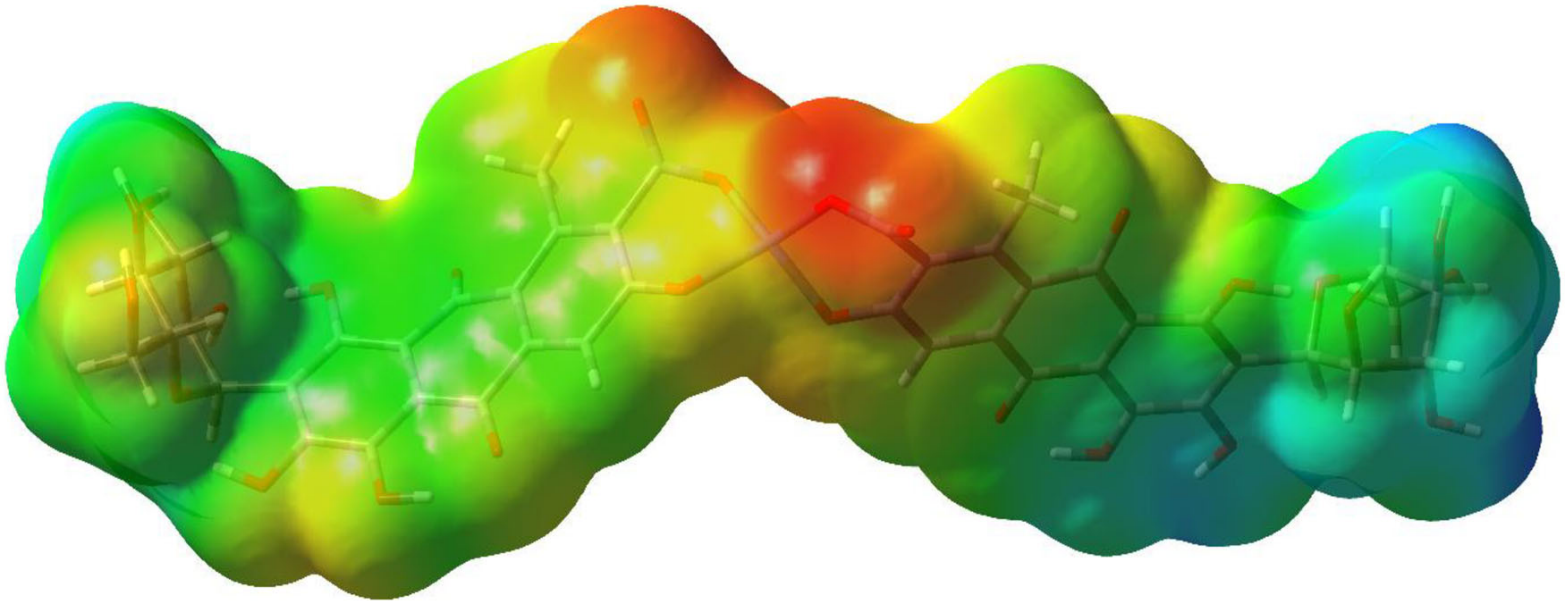
Molecular electrostatic potential map of Co(CA)_2_^2−.^

### 3.3 Interaction of Electrochemically Generated Species With Nucleobases

Both CA and Co(CA)_2_^2−^ when subjected to electrochemical reduction at their respective first reduction potential under deaerated (Ar saturated) conditions at pH ∼7.4, semiquinone is most likely the major product. However, other species also form. All such species would then interact with nucleobases maintained in the immediate vicinity of reduction of compounds. Aliquots collected were subjected to HPLC in order to ascertain the amount of a particular nucleobase remaining intact following interaction with reduced species in solution (Figure S9).

Modification of nucleobases were realized by plotting the amount of nucleobase remaining against the time for which reduction was performed on the compound, referred to as time for providing electrochemical stimuli (Figure 10A–E). Plots indicate degradation of nucleobases both in the absence and presence of sensitizers (compounds) under identical conditions. Slopes of degradation plots of nucleobases provide an estimate of the amount of damage caused to each of them. Different extents of base damage were observed for different nucleobases indicating some selectivity. This could be due to the type and/or amount of radicals generated in solution for CA and Co(CA)_2_^2−^. The extent of base damage depends upon the ability of reduced species to interact with a target (Figure 10, Table 2).

**FIGURE 10:**
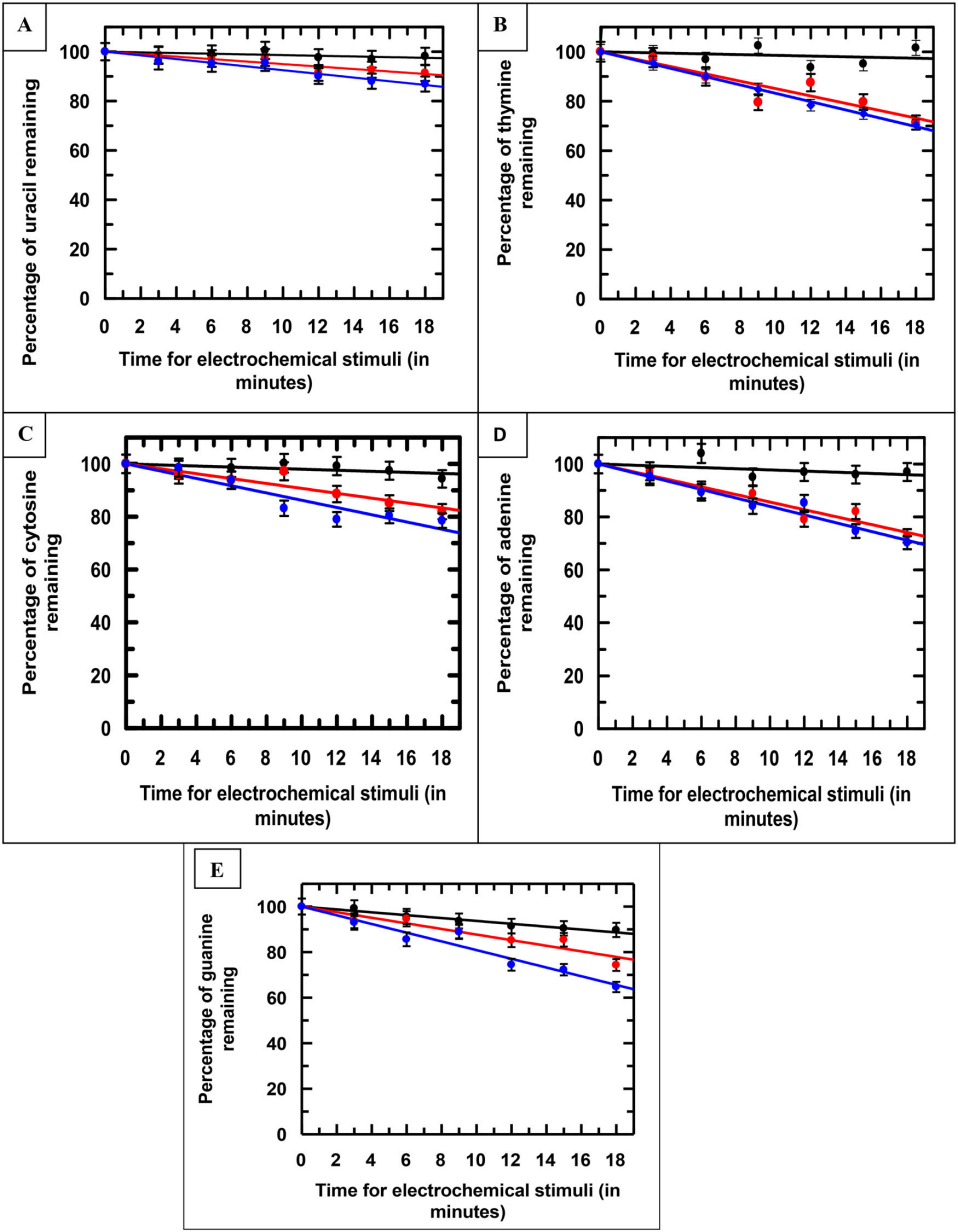
Plots for (A) uracil, (B) thymine, (C) cytosine, (D) adenine and (E) guanine are shown as targets. Each indicate percentage of that nucleobase remaining following interaction with in situ electrochemically generated species on CA and Co(CA)_2_^2−^. Different experimental conditions were (●) control; a nucleobase with no sensitizer added, (●) nucleobase in presence of CA as sensitizer, (●) nucleobase in presence of Co^II^(CA)_2_ as sensitizer.

**TABLE 2:** Results of degradation of nucleobases due to electrochemically generated species (semiquinone or other oxygen‐based radicals) in solution, followed by HPLC.

Compound as Sensitizer	T A R G E T											
	Uracil		Thymine		Cytosine		Adenine		Guanine		Calf thymus DNA	
	Slope of plot in de‐ aerated medium	ER	Slope of plot in deaerated medium	ER	Slope of plot in deaerated medium	ER	Slope of plot in deaerated medium	ER	Slope of plot in deaerated medium	ER	Slope of plot in deaerated medium	ER
	−0.138		−0.144		−0.183	—	−0.267	—	−0.631	—	−0.143	—
**CA**	−0.486	**3.5**	−1.59	**11.0**	−0.85	**4.6**	−1.18	**4.4**	−1.78	**1.7**	−0.26	**1.8**
**[Co(CA)_2_]^2−^**	−1.14	**8.2**	−1.41	**9.8**	−1.37	**7.5**	−1.24	**4.6**	−1.91	**3.0**	−0.40	**2.8**

Table 2 indicates in the absence of either CA or Co(CA)_2_^2−^, if a glassy carbon electrode was held at a potential intermediate between −0.65 V and −0.70 V, damage of nucleobases was almost negligible. However, when a sensitizer (compound) was introduced and a constant potential applied [−0.65 V for CA and −0.70 V for [Co(CA)_2_]^2−^], damage of nucleobases was observed suggesting reduced species formed on a compound react with a target maintained in immediate vicinity of the generation of such species. Damage caused to a nucleobase is expressed as Enhancement Ratio (ER), where ER is a value obtained by dividing the slope of a degradation plot achieved for a certain nucleobase in the presence of a sensitizer under a specific condition by the slope obtained for the damage of the same nucleobase in absence of any sensitizer. It is realized from Figure 10 and Table 2 that except for guanine, in the absence of a sensitizer no other nucleobase shows degradation following subjecting it to electrochemical stimuli for a specified time. Degradation plots for uracil (U), cytosine (C), thymine (T), and adenine (A) were almost in line with the 100 mark on the axis denoting “percentage of nucleobase remaining” for all plots.

Guanine (G) was found to be more susceptible to degradation under aforementioned conditions. This trend for damage observed for G was seen even in the presence of sensitizers CA or Co(CA)_2_^2−^ (Figure 10E). From Figure 10 and Table 2, it may be said apart from T and A where damage caused to a target was similar, be it in presence of CA or Co(CA)_2_^2−^, for all other nucleobases [U, C and G], damage observed in the presence of Co(CA)_2_^2−^ was much greater than that in presence of CA.

Hence, degradation of nucleobases, whether due to electrochemical stimuli induced radical pathway or that due to physical interaction between a sensitizer and a target [77], modifications on A and T were not only significantly greater than other nucleobases [U, C and G] but they were equal in magnitude (whether performed in presence of CA or in presence of Co(CA)_2_^2−^) (Figure 10, Table 2).

On the other hand, for U, C and G, results were clearly different; first, their damage was comparatively less than A and T and second, modifications in presence of Co(CA)_2_^2−^ was much more than in presence of CA. Table 2 provides a clear representation of the extent of damage caused to each nucleobase investigated as a part of this study. Results in Table 2 are an outcome of at least four separate experiments performed on a certain nucleobase. It should be mentioned here that since diffusion coefficients (D_0_) for CA and Co(CA)_2_^2−^ are different (D_0_ for CA and Co(CA)_2_^2−^ being 7.29 × 10^−8^ cm^2^/sec and 6.76 × 10^−6^ cm^2^/s, respectively) hence theoretically at least, there ought to be a difference in radical generation at the electrode‐solution interface; being somewhat more for Co(CA)_2_^2−^. This could be a reason for the better performance of the complex.

From ER values (Table 2) on the performance of CA and Co(CA)_2_^2−^ in electrochemical pathway for which monitoring of nucleobases were done, it might become possible to predict a damage that might occur due to such compounds on a particular type of DNA if its A‐T and G‐C compositions are known. Considering calf thymus DNA as an experimental target and having mole % G‐C = 41.9 and mole % A‐T = 58.1 [78, 79], an estimate based on our study on nucleobases (Table 2) would predict an ER of 5.79 for CA used as sensitizer and an ER of 6.38 for Co(CA)_2_^2−^ used as sensitizer.

However, since in cells, DNA is protected by chromatin organization, associated proteins, and active repair mechanisms within a microenvironment very different from an electrochemical system that we employed here, conclusions made from this study on model nucleobases and DNA should be considered with much greater caution.

### 3.4 Interaction of Electrochemically Generated Species With Calf Thymus DNA

CA and Co(CA)_2_^2−^ were subjected to electrochemical reduction in two separate experiments in presence of calf thymus DNA. Reduced species along with other products generated in solution were allowed to interact with calf thymus DNA (target), maintained in immediate vicinity of the generation of reduced species. Allowing a time interval of 5 min to 45 min, aliquots were withdrawn from an experimental solution on which a constant potential was applied either in absence or in presence of a sensitizer. Samples had similar concentrations of CA and Co(CA)_2_^2−^ to those used for studies with nucleobases. Double strand modification of calf thymus DNA was ascertained by treating aliquots withdrawn at different times from the reaction vessel with EtBr and exciting them at 510 nm. A decrease in fluorescence of the DNA‐EtBr adduct for an aliquot that was withdrawn, on which electrochemical stimuli was provided for a specific time (Figure 11) with respect to the situation where no electrochemical stimuli was provided, helped us to ascertain the extent of modification caused on DNA due to reduced species in solution.

**FIGURE 11:**
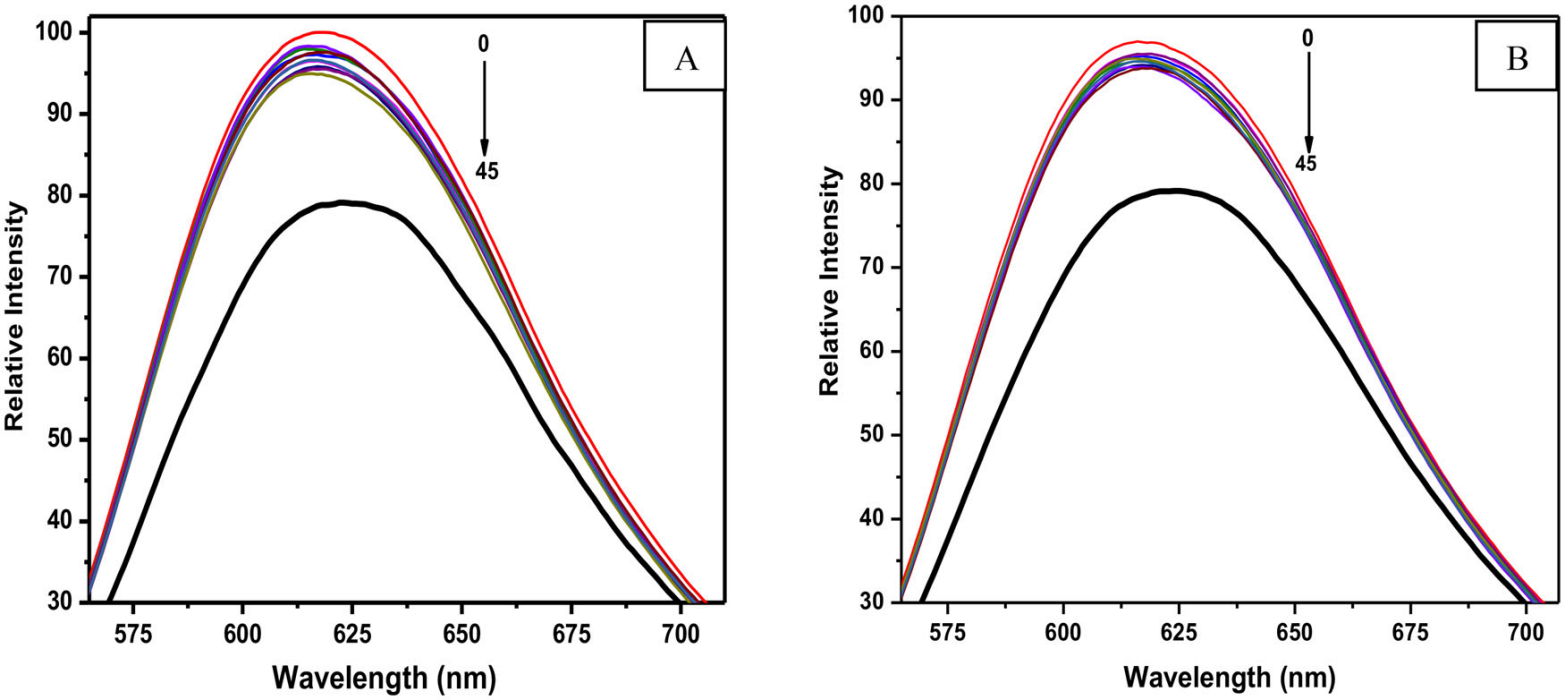
Fluorescence spectra of calf thymus DNA (44 µM) recorded after treatment of EtBr following its interaction with reduced products generated when (A) CA was subjected to reduction at a constant potential of −0.65 V and (B) when Co(CA)_2_^2−^ was subjected to reduction at a constant potential of −0.70 V in deaerated (Argon saturated) condition. [CA] = [Co(CA)_2_^2−^] = 4.5 µM. “0” to “45” indicates time in minutes for which constant potential was applied; black lines in both figures denote the spectrum of EtBr when it was excited alone, that is, in the absence of DNA at 520 nm.

Figure 12 shows the amount of calf thymus DNA remaining intact following interaction with reactive intermediates (semiquinone/protonated semiquinone or other ROS) formed in solution at pH 7.4. It indicates double strand modification of calf thymus DNA was higher when Co^II^(CA)_2_^2−^ is present in the medium than when CA is present (Table 2). Interestingly, the damage actually observed for calf thymus DNA (Figure 12) had the same trend as that observed for nucleobases following interaction with species generated under identical conditions. The study on calf thymus DNA shows ER obtained experimentally for both compounds were actually half or even lower than that predicted on the basis of studies on nucleobases. Prediction of ER for calf thymus DNA on the basis of nucleobase composition for CA as sensitizer was 5.79 while that found experimentally was 1.8; for Co^II^(CA)_2_^2−^ prediction was 6.38 and that found experimentally was 2.8. Although the trend for ER obtained experimentally was the same as that of the prediction, findings clearly indicate that a study performed on isolated nucleobases might not be able to provide a correct picture; this could be because in case of DNA, the same nucleobases that we worked with are now connected to sugar and phosphates that are in turn interconnected to each other, resulting in a polymeric structure that provide stability compared to isolated nucleobases, when subjected to free radical attack. This study thus indicates that it might be difficult to damage interconnected nucleobases as in DNA than isolated ones.

**FIGURE 12:**
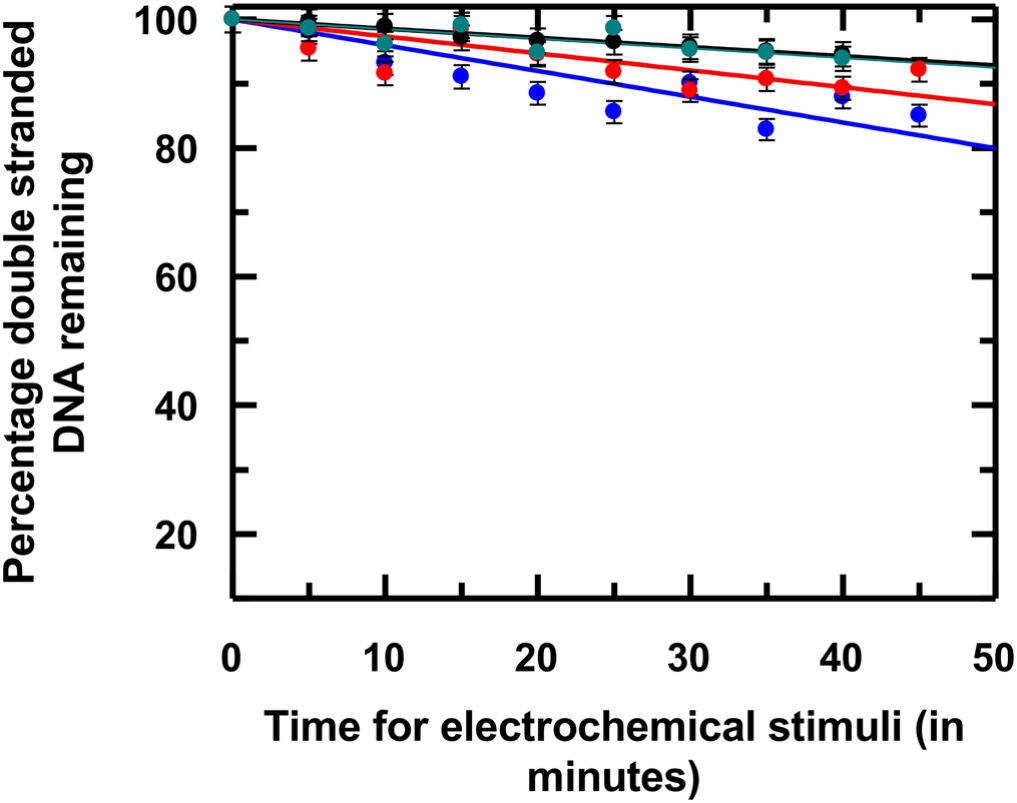
Plots show modification of calf thymus DNA in the absence of any compound (●), in presence of Co^II^Cl_2_ (●); in the presence of CA (●) and Co^II^(CA)_2_^2−^ (●) in an argon saturated medium at pH 7.4; [DNA] = 45 µM; [Co^II^Cl_2_] = [CA] = [Co^II^(CA)_2_^2−^] = 4.5 µM.

Besides, in the study with DNA, apart from interaction with various reactive oxygen species formed in solution there would also be the aspect of physical interaction between compounds and DNA, either by intercalation or groove binding that should also contribute to modification. These would also be detected in ethidium bromide‐fluorescence technique used. Hence, in case of a study performed with DNA in the absence of electrochemical stimuli there would be substantial modification that was not the case for isolated nucleobases except for guanine. Therefore, it should be kept in mind that the very basis for calculation of ER is different for the two targets (nucleobases and DNA). While for DNA, it would be essential to consider damage owing to physical interaction besides that due to free radical pathway, for nucleobases, the aspect of physical interaction would be negligible [80]. Besides, study on nucleobases was on targets that remain isolated in aqueous solution, for DNA nucleobases are bound to each other via phosphate and sugar providing extra stability. This could be a reason for observing decreased damage under almost identical experimental conditions implying interaction with isolated nucleobases and interconnected nucleobases are different. However, since the experimental trend observed for calf thymus DNA was the same as that for a prediction based on a study performed on nucleobases (for both compounds), this would indicate that distortion and/or modification of DNA might have originated at a nucleobase site. This would obviously be better realized if studies are carried out on nucleosides or oligonucleotides. However, since that wasn't done as a part of this study, hence, at this point, more than what is mentioned above should not be claimed regarding interactions between compounds used and DNA. A recent study indicated DNA and oligonucleotides stabilize semiquinone radical anion by delocalization of electrons in a π‐stacking framework resulting in a radical intercalated situation rather than formation of covalent bonds [80]. A semiquinone radical anion present in a DNA strand has also been reported to cause strand breaks that could result in disruption of several processes in which DNA is involved [78, 79, 80]. Since our studies show modification of nucleobases, hence, if there is intercalation due to compounds that result in unwinding of DNA, then exposed nucleobases could become vulnerable to damage, that is, permanently preventing them from regenerating a double helical structure again. If this happens, such activity could well be realized using the “ethidium bromide‐fluorescence” technique and such compounds could be used as chemotherapeutic agents. To be sure about the role of Co^II^ in the complex we carried out the same study in presence of aq. Co^2+^ (procured from CoCl_2_.6H_2_O). Results show that the black line (for control experiment) in Figure 12 and green line when the experiment was carried out in presence of Co^II^ were actually identical. Hence, we may say Co^II^ when present in the complex could have a role in DNA modification than when simply taken as a salt, thus justifying its presence in the complex.

### 3.5 Assessment of Radical Formation on CA Through Scavenging of DPPH Using EPR Spectroscopy

To establish that a free radical was formed on CA following its electrochemical reduction, a DPPH radical scavenging study was done [65, 66, 67, 81, 82]. For the same initial concentration of DPPH (6 mM, 500 µL), Figure 13 very clearly shows that the signal for DPPH in presence of electrochemically reduced CA was much smaller than for DPPH alone (i.e., in absence of electrochemically reduced CA). This decrease in EPR response suggests a significant amount of the radical formed on CA is no longer present. Conversion of DPPH• to reduced DPPH upon interaction with semiquinone radical anion (Q^•−^) suggests it acts as a powerful reducing agent. Interaction results in electron or hydrogen atom transfer neutralizing both radicals and permitting one to follow radical‐scavenging activity.

**FIGURE 13:**
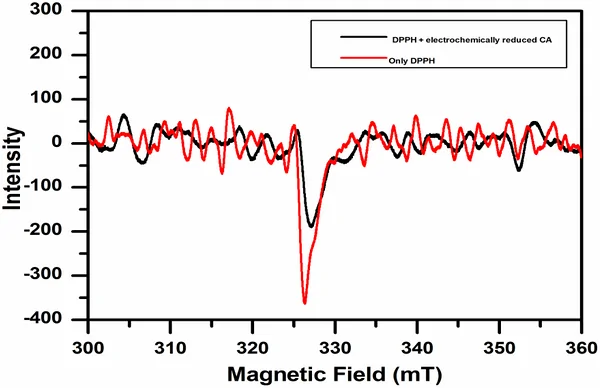
EPR spectra of DPPH alone (6 mM, 500 µL; in red) and that in the presence of electrochemically reduced CA (in black). Ethanol was used as solvent.

In case of electron transfer, Q^•−^ donates an electron to DPPH• forming DPPH anion (DPPH^−^), itself getting converted to quinone (Q):

(6) $$Q^{\cdot -}+{\mathrm{DPPH}}^{\cdot }\to Q+{\mathrm{DPPH}}^{-}$$

For proton‐coupled electron transfer, DPPH^−^ captures a proton from the solvent to form stable diamagnetic hydrazine (DPPH‐H).

(7) $${\mathrm{DPPH}}^{-}+H^{+}\to \mathrm{DPPH}-H$$

Whichever happens, we can conclude a semiquinone must have formed on CA following its electrochemical reduction for it to be able to quench the free radical (DPPH•) in solution.

### 3.6 Detection of ROS by DCFDA Assay

Figure 14 demonstrates there is significant presence of ROS in the medium of PC3 (human prostate carcinoma) cells under different experimental conditions. One may also see that for different compositions of the medium like that having H_2_O_2_ or CA or [Co^II^(CA)_2_]^2−^ or CA with H_2_O_2_ or [Co^II^(CA)_2_]^2−^with H_2_O_2_, presence of ROS in the medium is sufficiently high depending on what is present, against a situation where no compound is used (i.e., presence of ROS is due to cellular metabolism only). It is seen that when Co^II^(CA)_2_^2−^ is present in cellular medium, presence of ROS increased to proportions much higher than when the complex is not present suggesting a possible role for the Co^II^/Co^III^ couple; NAC pre‐treatment significantly reduced this fluorescence, confirming a ROS‐dependent induction. Therefore, H_2_DCFDA assay performed on PC3 cells indicates why the complex performs better and that the free radical pathway might have played a dominant role; a fact also realized through studies carried on model nucleobases and calf thymus DNA.

**FIGURE 14:**
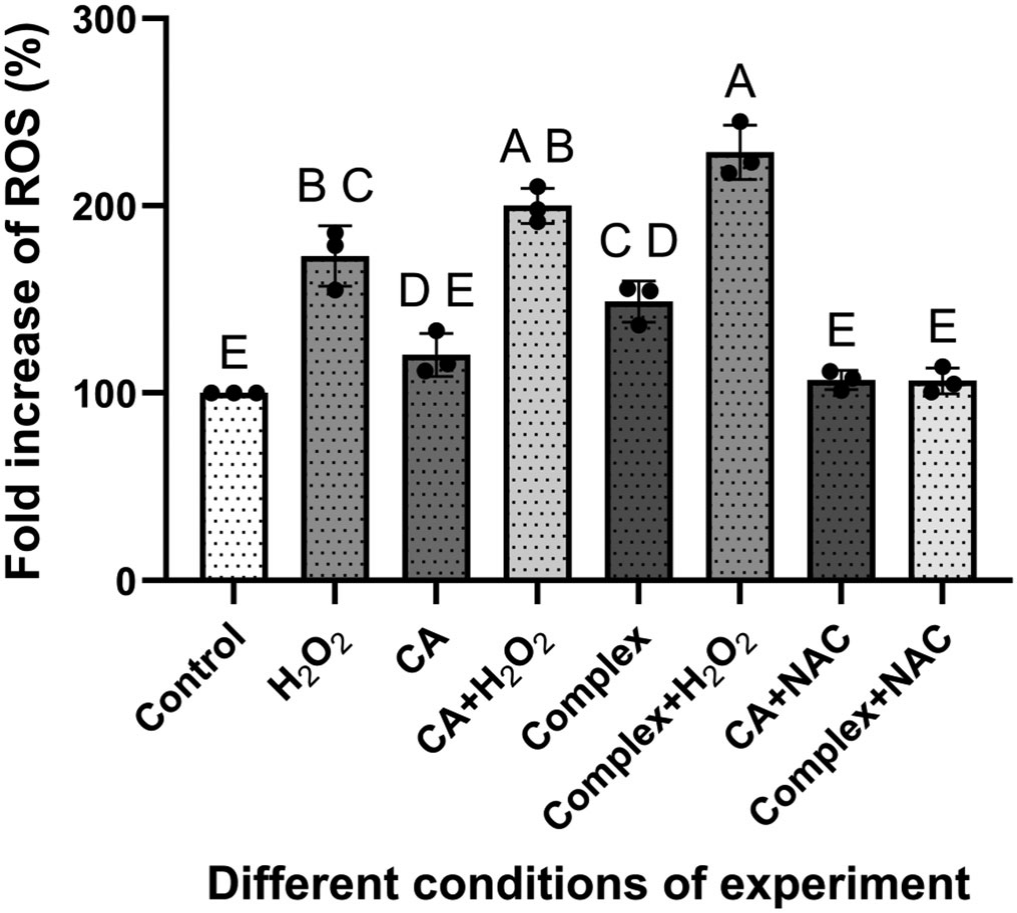
Effect of different compounds/conditions of experiments on the generation of ROS in PC3 (human prostate carcinoma cells) that was followed by H_2_DCFDA assay. Bars represent mean ± SD. Different letters indicate statistically significant differences (*p* < 0.05); groups sharing the same letter are not significantly different.

Figure 15 shows the influence of concentration of compounds [CA and Co^II^(CA)_2_^2−^] on the generation of ROS in cellular medium. It very clearly demonstrates that in case of the complex, with increase in concentration there is an increase in generation of ROS that has an impact on target cells when such compounds would be used as agents of cellular destruction.

**FIGURE 15:**
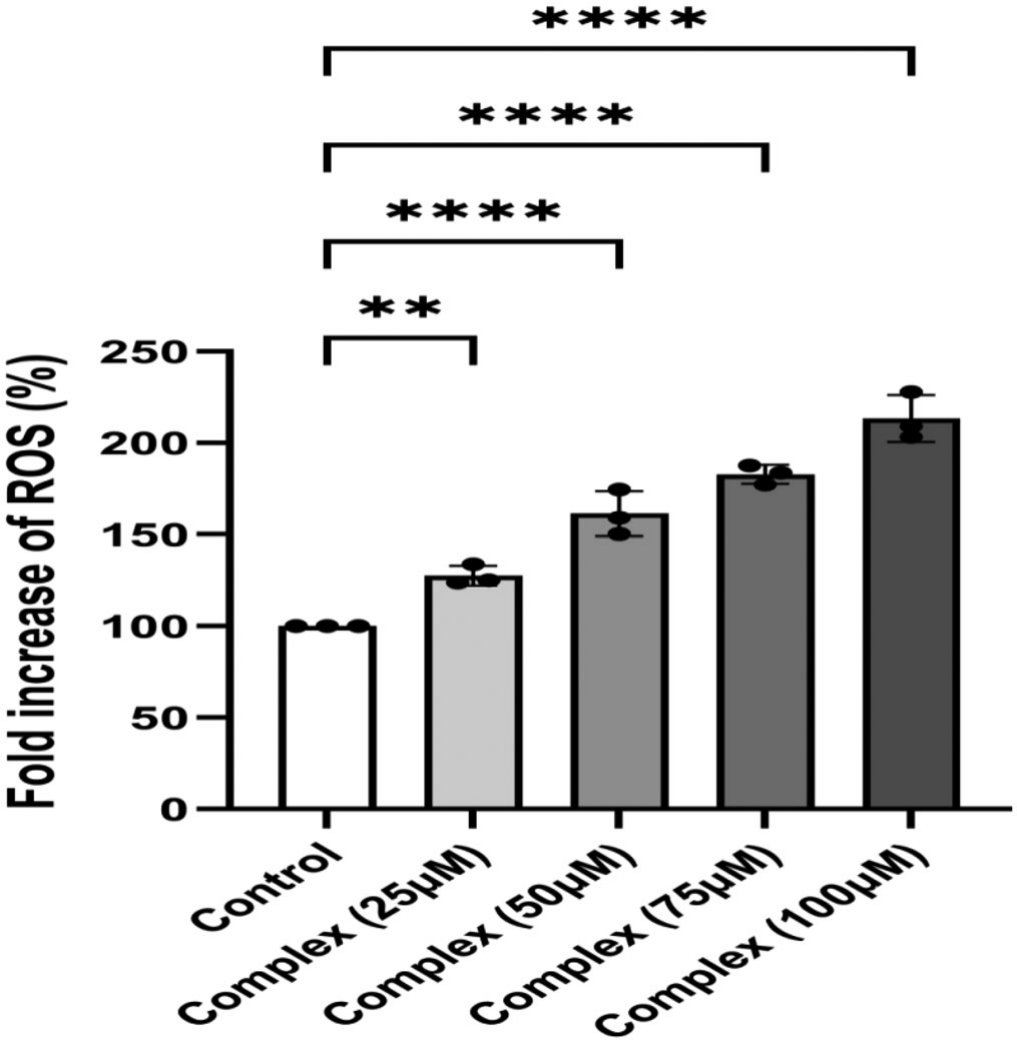
Dose‐dependent induction of intracellular ROS in PC3 cells. PC3 cells were treated with increasing concentrations of sample for 1 h, followed by incubation with H_2_DCFDA. Intracellular ROS levels were quantified as fluorescence intensity and normalized to vehicle control (^****^ *p* < 0.0001). Data are presented as mean ± SD (*n* = 3 independent experiments).

Although Co^II^(CA)_2_^2−^ significantly reduced cell viability and increased intracellular oxidative stress, the present study did not determine the specific mode of cell death. Additional investigations using Annexin V/PI staining, caspase activation assays, mitochondrial membrane potential analysis (JC‐1), and other pathway‐specific assays would be required to distinguish apoptosis, necrosis, ferroptosis or other mechanisms.

### 3.7 Cell Viability Assay

Cytotoxicity related investigations due to CA and [Co^II^(CA)_2_]^2−^ were carried out on PC3 human prostate carcinoma and WI 38 normal lung fibroblast cells using MTT assay. Findings are based on interaction of chosen cells with different concentrations of CA and [Co^II^(CA)_2_]^2−^, able to predict a cell survival of 50% (IC_50_) in comparison to untreated cells. IC_50_ for CA and [Co^II^(CA)_2_]^2−^ on PC3 human prostate carcinoma cells were 43.0 µM and 32.0 µM respectively (Figure 16) indicating the complex to be more effective. On the other hand, on WI 38 cells, IC_50_ for CA was 240 µM while that for [Co^II^(CA)_2_]^2−^ was 200 µM (Figure S10). Both Figure 16 and Figure S10 seem to suggest compounds CA and [Co^II^(CA)_2_]^2−^ are more effective on carcinoma cells than normal cells. However, a limitation to the present study are issues related to the stability of [Co^II^(CA)_2_]^2−^ in cell culture medium as that could not be evaluated separately (to enable one to say whether there was any dissociation of [Co^II^(CA)_2_]^2−^; by way of evaluation of either free CA or [Co^II^CA] or any other probable species). However, as part of future investigations, we would perform a physicochemical stability of the complex under physiological conditions to better correlate its structural integrity with biological activity.

**FIGURE 16:**
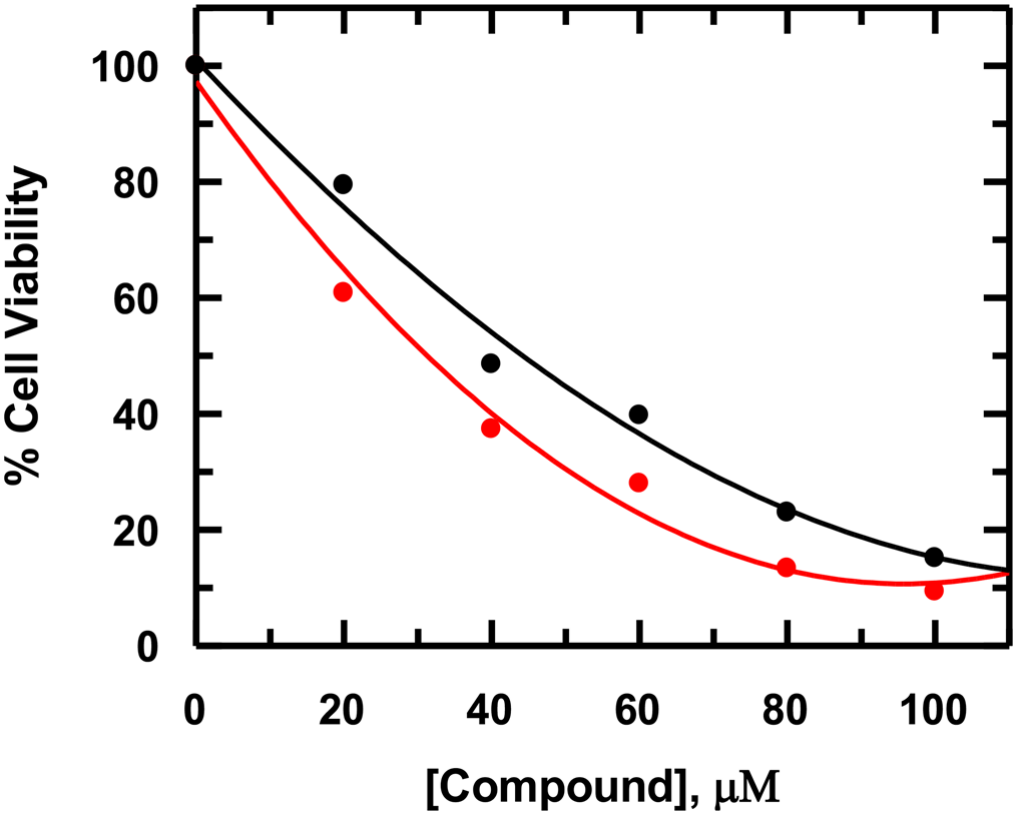
Cytotoxic effect of CA (—•—) and [Co^II^(CA)_2_]^2−^ (—•—) on PC3 human prostate carcinoma cells following exposure for 24 h.

## 4 Conclusion

Carminic acid resembles the anthracycline class of antibiotics very closely. In this study, it was chosen with a view to realize its potential as an anticancer agent. Although essentially a hydroxy‐9,10‐anthraquinone, it is biologically more viable as it has a sugar unit in its structure. To equip it further with attributes that are essential for anticancer activity, that is, besides what it already possesses, and to try to improve its efficacy in targeting biological environments, a coordination compound with Co^II^ was prepared. The compound was characterized using different spectroscopic techniques and mass spectrometry. Using DFT and magnetic susceptibility measurements, the structure was found to be slightly distorted square planar. Carminic acid and its Co^II^ complex were subjected to electrochemical reduction using a potentiostat in the presence of nucleobases and calf thymus DNA to investigate their role in a free radical pathway, believed to be significant by which anthracyclines or its analogs show cytotoxic activity on cancer cells. The study demonstrates how nucleobases that constitute DNA might be affected due to interaction with semiquinone‐radical anion and/or other reduced species formed on compounds following their reduction in aqueous solution. The study demonstrates a compound's ability to induce damage on chosen nucleobases. The study showed Co^II^ complex of carminic acid was more effective on nucleobases like uracil, cytosine, guanine and on calf thymus DNA than carminic acid itself. This was supported by another study that utilized the H_2_DCFDA assay performed on PC3 carcinoma cells. Activity of both compounds was checked on PC3 human prostate carcinoma and WI‐38 normal human fetal lung fibroblast cells. Both CA and [Co^II^(CA)_2_]^2−^ were more active on carcinoma cells than on normal cells. In case of the carcinoma cell line, Co^II^ complex was more effective than CA. As identified earlier for several complexes of anthracyclines or of hydroxy‐9,10‐anthraquinones, there is observed a decrease in semiquinone generation following complex formation [32, 33, 34, 35, 36]. If that is the trend, it should be true for this complex also. In that case, one expects a loss in efficacy for the complex owing to decrease in semiquinone formation (implying there would be decrease in superoxide radical‐anion also) [29, 30, 31, 32, 33, 34, 35, 36, 41, 42, 43, 44, 45, 46, 47]. Instead, since efficacy on nucleobases and on DNA was found to be enhanced in case of the complex than for CA itself, hence, several other properties incorporated probably compensate the loss in activity of the complex in a free radical pathway to be able to see such results. Moreover, what is more important is that the complex should have a reduced risk of cardiotoxicity, a worrying aspect for anthracyclines. Hence, with increased efficacy, reduced cardiotoxic side effects, a much lower cost, a Co^II^ complex of carminic acid seems to have a lot of promise as a viable alternative to anthracyclines in cancer chemotherapy. However, there are certain limitations to this study: (i) an absence of aqueous Cobalt^II^‐only control (aqueous Cobalt^II^ chloride) for the MTT assay which could have helped to distinguish between effects arising from free cobalt^II^ ions and those specific to [Co^II^(CA)_2_]^2−^ (the complex), (ii) that the present study is unable to say with certainty whether [Co^II^(CA)_2_]^2−^ in cell culture medium stays intact as one unit or dissociates to different species; whether the observed effects are due to [Co^II^(CA)_2_]^2−^ or a cumulative effect of it and its dissociated species, and (iii) “mimicking the real cellular environment” through model experiments was certainly not exactly possible; our study at best may be said to be an useful comparative chemical model but not a direct mechanistic simulation of intracellular behavior and uncontrolled biological variables that may lead to substantially different cellular behavior.

## Conflicts of Interest

The authors declare no conflicts of interest.

## Supporting information




**Supporting File 1**: cbdv71855‐sup‐0001‐SuppMat.docx.

## Data Availability

The data that support the findings of this study are available in the supplementary material of this article.

## References

[1] J. V. McGowan , R. Chung , A. Maulik , I. Piotrowska , J. M. Walker , and D. M. Yellon , “Anthracycline Chemotherapy and Cardiotoxicity,” Cardiovascular Drugs and Therapy 31 (2017): 63–75, 10.1007/s10557-016-6711-0. PMC5346598 28185035

[2] D. W. Edwardson , R. Narendrula , S. Chewchuk , K. Mispel‐Beyer , J. P. J. Mapletoft , and A. M. Parissenti , “Role of Drug Metabolism in the Cytotoxicity and Clinical Efficacy of Anthracyclines,” Current Drug Metabolism 16 (2015): 412–426, 10.2174/1389200216888150915112039. PMC5398089 26321196

[3] R. Mattioli , R. A. Ilari , B. Colotti , L. Mosca , F. Fazi , and G. Colotti , “Doxorubicin and Other Anthracyclines in Cancers: Activity, Chemoresistance and Its Overcoming,” Molecular Aspects of Medicine 93 (2023): 101205, 10.1016/j.mam.2023.101205. 37515939

[4] H. Wang , X. Xiao , Q. Xiao , Y. Lu , and Y. Wu , “The Efficacy and Safety of Daunorubicin Versus Idarubicin Combined With Cytarabine for Induction Therapy in Acute Myeloid Leukemia,” Medicine 99, no. 24 (2020): e20094, 10.1097/MD.0000000000020094. PMC7302600 32541448

[5] S. Y. van der Zanden , X. Qiao , and J. Neefjes , “New Insights Into the Activities and Toxicities of the Old Anticancer Drug Doxorubicin,” FEBS Journal 288 (2021): 6095–6111. 10.1111/febs.15583 PMC8597086 33022843

[6] W. C. M. Dempke , R. Zielinski , C. Winkler , S. Silberman , S. Reuther , and W. Priebe , “Anthracycline‐Induced Cardiotoxicity — Are We About to Clear this Hurdle?,” European Journal of Cancer 185 (2023): 94–104, 10.1016/j.ejca.2023.02.019. 36966697

[7] L. A. Smith , V. R. Cornelius , C. J. Plummer , et al., “Cardiotoxicity of Anthracycline Agents for the Treatment of Cancer: Systematic Review and Meta‐Analysis of Randomised Controlled Trials,” BMC Cancer 10 (2010): 337, 10.1186/1471-2407-10-337. PMC2907344 20587042

[8] D. Cardinale , F. Iacopo , and C. M. Cipolla , “Cardiotoxicity of Anthracyclines,” Frontiers in Cardiovascular Medicine 7 (2020): 26, 10.3389/fcvm.2020.00026. PMC7093379 32258060

[9] W. Owattanapanich , N. Owattanapanich , S. Kungwankiattichai , P. Ungprasert , and T. Ruchutrakool , “Efficacy and Toxicity of Idarubicin Versus High‐Dose daunorubicin for Induction Chemotherapy in Adult Acute Myeloid Leukemia: A Systematic Review and Meta‐Analysis,” Clinical Lymphoma Myeloma and Leukemia 18 (2018): 814–821.e3, 10.1016/j.clml.2018.08.008. 30241991

[10] C. G. Nebigil and L. Désaubry , “Updates in Anthracycline‐Mediated Cardiotoxicity,” Frontiers in Pharmacology 9 (2018): 1262, 10.3389/fphar.2018.01262. PMC6240592 30483123

[11] R. Adão , G. de Keulenaer , A. Leite‐Moreira , and C. Brás‐Silva , “Cardiotoxicity Associated With Cancer Therapy: Pathophysiology and Prevention,” Revista Portuguesa De Cardiologia 32 (2013): 395–409. 10.1016/j.repc.2012.11.002 23623503

[12] X. Han , Y. Zhou , and W. Liu , “Precision Cardio‐Oncology: Understanding the Cardiotoxicity of Cancer Therapy,” npj Precision Oncology 1 (2017): 31, 10.1038/s41698-017-0034-x. PMC5871905 29872712

[13] S. E. Lipshultz , J. A. Alvarez , and R. E. Scully , “Anthracycline Associated Cardiotoxicity in Survivors of Childhood Cancer,” Heart 94 (2008): 525–533, 10.1136/hrt.2007.136093. 18347383

[14] Y. Kwon , “Mechanism‐Based Management for Mucositis: Option for Treating Side Effects Without Compromising the Efficacy of Cancer Therapy,” OncoTargets and Therapy 9 (2016): 2007, 10.2147/OTT.S96899. PMC4827894 27103826

[15] P. A. Zulfugarova , S. I. Humbatov , and A. A. Mekhtiev , “Molecular Mechanisms of Adaptation to Desynchronization Stress,” Advances in Biology & Earth Sciences 10 (2025): 621–628.

[16] L. Huseynova , “Pyruvate Kinase Modulation in the Brain Under Stress Factors: Structural, Developmental and Molecular Perspectives,” Advances in Biology & Earth Sciences 10 (2025): 488–498.

[17] R. S. Cvetkovie and L. J. Scott , “Dexrazoxane: A Review of Its Use for Cardioprotection During Anthracycline Chemotherapy,” Drugs 65 (2005): 1005–1024, 10.2165/00003495-200565070-00008. 15892593

[18] Y. Pommier , E. Leo , H. L. Zhang , and C. Marchand , “DNA Topoisomerases and Their Poisoning by Anticancer and Antibacterial Drugs,” Chemistry & Biology 17 (2010): 421–433, 10.1016/j.chembiol.2010.04.012. PMC7316379 20534341

[19] P. Das , C. K. Jain , S. K. Dey , et al., “Synthesis, Crystal Structure, DNA Interaction and In Vitro Anticancer Activity of a Cu(II) Complex of Purpurin: Dual Poison for Human DNA Topoisomerase I and II,” RSC Advances 4 (2014): 59344–59357, 10.1039/C4RA07127A.

[20] P. Das , C. K. Jain , S. Roychoudhury , H. K. Majumder , and S. Das , “Design, Synthesis and In Vitro Anticancer Activity of a Cu(II) Complex of Carminic Acid: A Novel Small Molecule Inhibitor of Human DNA Topoisomerase I and Topoisomerase II,” ChemistrySelect 1 (2016): 6623–6631, 10.1002/slct.201601152.

[21] I. A. Riddell , K. Agama , G. Y. Park , Y. Pommier , and S. J. Lippard , “Phenanthriplatin Acts as a Covalent Poison of Topoisomerase II Cleavage Complexes,” ACS Chemical Biology 11 (2016): 2996–3001, 10.1021/acschembio.6b00565. PMC5248983 27648475

[22] B. Pang , X. Qiao , L. Janssen , et al., “Drug‐Induced Histone Eviction From Open Chromatin Contributes to the Chemotherapeutic Effects of Doxorubicin,” Nature Communications 4 (2013): 1908, 10.1038/ncomms2921. PMC3674280 23715267

[23] M. A. van Gelder , S. Y. van der Zanden , M. B. L. Vriends , et al., “Re‐Exploring the Anthracycline Chemical Space for Better Anti‐Cancer Compounds,” Journal of Medicinal Chemistry 66 (2023): 11390–11398. 10.1021/acs.jmedchem.3c00853 PMC10461226 37561481

[24] X. Qiao , S. Y. van der Zanden , and D. P. A. Wander , “Uncoupling DNA Damage From Chromatin Damage to Detoxify Doxorubicin,” Proceedings of the National Academy of Sciences 117 (2020), 15182–15192, 10.1073/pnas.1922072117. PMC7334570 32554494

[25] M. Javan , M. Ebrahimi , A. Madatli , F. Madatli , and R. Karimova , “Acetylation and Sirtuins: Molecular Mechanisms Driving Metabolic Flexibility,” Advances in Biology & Earth Sciences 10 (2025): 547–584.

[26] J.‐M. Nabholtz and A. Riva , “Taxane/Anthracycline Combinations: Setting a New Standard in Breast Cancer?” Oncologist 6 (2001): 5–12, 10.1634/theoncologist.6-suppl_3-5. 11346678

[27] Y. Sun , “Chemosensitization by Emodin, a Plant‐Derived Anti‐Cancer Agent: Mechanism of Action,” Cancer Biology & Therapy 7 (2008): 476–478, 10.4161/cbt.7.3.5584. 18245955

[28] X. Huang , J. Wang , C. Huang , et al., “Emodin Enhances Cytotoxicity of Chemotherapeutic Drugs in Prostate Cancer Cells: The Mechanisms Involve ROS‐Mediated Suppression of Multidrug Resistance and Hypoxia Inducible Factor‐1,” Cancer Biology & Therapy 7 (2008): 468–475. 10.4161/cbt.7.3.5457 18285700

[29] M. Shandilya , S. Sharma , P. P. Das , and S. Charak , “Molecular‐Level Understanding of the Anticancer Action Mechanism of Anthracyclines,” in Advances in Precision Medicine Oncology, edited by H. Arnouk and B. Hassan (IntechOpen, 2020), Open Access Peer‐Reviewed Edited Volume, 10.5772/intechopen.94180.

[30] S. Mukherjee , P. K. Gopal , S. Paul , and S. Das , “Acetylation of 1, 2, 5, 8‐Tetrahydroxy‐9, 10‐Anthraquinone Improves Binding to DNA and Shows Enhanced Superoxide Formation That Explains Better Cytotoxicity on JURKAT T Lymphocyte Cells,” Journal of Analytical Oncology 3 (2014): 122–129.

[31] J. L. Bolton and T. Dunlap , “Formation and Biological Targets of Quinones: Cytotoxic Versus Cytoprotective Effects,” Chemical Research in Toxicology 30 (2017): 13–37, 10.1021/acs.chemrestox.6b00256. PMC5241708 27617882

[32] P. Das , D. Bhattacharya , P. Karmakar , and S. Das , “Influence of Ionic Strength on the Interaction of THA and Its Cu(II) Complex With DNA Helps to Explain Studies on Various Breast Cancer Cells,” RSC Advances 5 (2015): 73099–73111, 10.1039/C5RA07805A.

[33] B. Mandal , S. Singha , S. K. Dey , et al., “Synthesis, Crystal Structure From PXRD of a Mn^II^ (purp)_2_ Complex, Interaction With DNA at Different Temperatures and pH and Lack of Stimulated ROS Formation by the Complex,” RSC Advances 6 (2016): 51520–51532, 10.1039/C6RA09387F.

[34] B. Mandal , S. Singha , S. K. Dey , et al., “Cu^II^ Complex of Emodin With Improved Anticancer Activity as Demonstrated by Its Performance on HeLa and Hep G2 Cells,” RSC Advances 7 (2017): 41403–41418, 10.1039/C7RA06696A.

[35] S. Mukherjee Chatterjee , C. K. Jain , S. Singha , et al., “Activity of CoII–Quinalizarin: A Novel Analogue of Anthracycline‐Based Anticancer Agents Targets Human DNA Topoisomerase, Whereas Quinalizarin Itself Acts via Formation of Semiquinone on Acute Lymphoblastic Leukemia MOLT‐4 and HCT 116 Cells,” ACS Omega 3 (2018): 10255–10266, 10.1021/acsomega.8b00706. PMC6644896 31459155

[36] M. Saha , S. Singha , M. Chakraborty , et al., “Characterization of a Mn^II^ Complex of Alizarin Suggests Attributes Explaining a Superior Anticancer Activity: A Comparison With Anthracycline Drugs,” Polyhedron 173 (2019): 114104, 10.1016/j.poly.2019.08.007.

[37] A. Akbar , A. Jabbar Siddiqui , S. Tarique Moin , et al., “A Rapid Colorimetric Method for the Detection of Carminic Acid in Samples Based on Visible Color Change,” Spectrochimica Acta Part A: Molecular and Biomolecular Spectroscopy 302 (2023): 122953, 10.1016/j.saa.2023.122953. 37392539

[38] G. Pastuch‐Gawołek , J. Szreder , M. Domińska , M. Pielok , P. Cichy , and M. Grymel , “A Small Sugar Molecule With Huge Potential in Targeted Cancer Therapy,” Pharmaceutics 15 (2023): 913, 10.3390/pharmaceutics15030913. PMC10056414 36986774

[39] Í. P. de Souza , A. C. C. de Melo , B. L. L. Rodrigues , et al., “Antitumor Copper(II) Complexes With Hydroxyanthraquinones and N,N‐Heterocyclic Ligands,” Journal of Inorganic Biochemistry 241 (2023): 112121. 10.1016/j.jinorgbio.2023.112121 36696836

[40] M. Sakamaki , S. Aikawa , and Y. Fukushima , “Colorimetric Determination of Pb^2+^ in Perfect Aqueous Solution Using Carminic Acid as a Selective Chemosensor,” Journal of Fluorescence 27 (2017): 1929–1935, 10.1007/s10895-017-2131-1. 28634885

[41] P. S. Guin , S. Das , and P. C. Mandal , “Electrochemical Reduction of Sodium 1,4‐Dihydroxy‐9,10‐Anthraquinone‐2‐sulphonate in Aqueous and Aqueous Dimethyl Formamide Mixed Solvent: A Cyclic Voltammetric Study,” International Journal of Electrochemical Science 3 (2008): 1016–1028, 10.1016/S1452-3981(23)15500-3.

[42] P. Das , P. S. Guin , P. C. Mandal , M. Paul , S. Paul , and S. Das , “Cyclic Voltammetric Studies of 1,2,4‐Trihydroxy‐9,10‐Anthraquinone, Its Interaction With Calf Thymus DNA and Anti‐Leukemic Activity on MOLT‐4 Cell Lines: A Comparison With Anthracycline Anticancer Drugs,” Journal of Physical Organic Chemistry 24 (2011): 774–785, 10.1002/poc.1827.

[43] S. Banerjee , S. Roy , D. Dharumadurai , et al., “A Co(III) Complex of 1‐Amino‐4‐Hydroxy‐9,10‐Anthraquinone Exhibits Apoptotic Action Against MCF‐7 human Breast Cancer Cells,” ACS Omega 7 (2022): 1428–1436, 10.1021/acsomega.1c06125. PMC8756448 35036804

[44] B. Mandal , H. K. Mondal , and S. Das , “In Situ Reactivity of Electrochemically Generated Semiquinone on Emodin and Its Cu^II^/Mn^II^ Complexes With Pyrimidine Based Nucleic Acid Bases and Calf Thymus DNA: Insight Into Free Radical Induced Cytotoxicity of Anthracyclines,” Biochemical and Biophysical Research Communications 515 (2019): 505–509, 10.1016/j.bbrc.2019.05.140. 31171362

[45] M. Saha and S. Das , “Free Radical Induced Activity of an Anthracycline Analogue and Its Mn^II^ Complex on Biological Targets Through In Situ Electrochemical Generation of Semiquinone,” Heliyon 7 (2021): e07746, 10.1016/j.heliyon.2021.e07746. PMC8379465 34458604

[46] S. Chatterjee , C. K. Jain , T. Saha , S. Roychoudhury , H. K. Majumder , and S. Das , “Utilizing Coordination Chemistry Through Formation of a Cu^II^‐quinalizarin Complex to Manipulate Cell Biology: An *In Vitro*, *In Silico* Approach,” Journal of Inorganic Biochemistry 249 (2023): 112369, 10.1016/j.jinorgbio.2023.112369. 37776829

[47] M. Saha , S. Singha , D. Ghosh , S. Kumar , P. Karmakar , and S. Das , “A Cobalt^II^/Cobalt^III^ Complex of Alizarin That Was Analyzed From the Stand Point of Binding With DNA, for ROS Generation and Anticancer Drug Prospecting Was Identified as an Analogue of Anthracyclines,” Journal of Molecular Structure 1262 (2022): 133011, 10.1016/j.molstruc.2022.133011.

[48] B. Xu , Z. Luo , T. Tao , and Y. Wang , “Dual Acceleration of Co(III)/Co(II) Redox Cycle Boosts Peroxymonosulfate Activation for Superfast Chloramphenicol Degradation,” Separation and Purification Technology 334 (2024): 126321, 10.1016/j.seppur.2024.126321.

[49] H. Mizutani , A. Nishimoto , S. Hotta , et al., “Oxidative DNA Damage Induced by Pirarubicin, an Anthracycline Anticancer Agent, in the Presence of Copper(II),” Anticancer Research 38 (2018): 2643–2648. 10.21873/anticanres.12506 29715084

[50] X. Y. Wang , I. Yokoi , J. K. Liu , and A. Mori , “Cobalt(II) and Nickel(II) Ions as Promoters of Free Radicals *In Vivo*: Detected Directly Using Electron Spin Resonance Spectrometry in Circulating Blood in Rats,” Archives of Biochemistry and Biophysics 306 (1993): 402–406, 10.1006/abbi.1993.1529. 8215442

[51] B. Scharf , C. C. Clement , V. Zolla , et al., “Molecular Analysis of Chromium and Cobalt‐Related Toxicity,” Scientific Reports 4 (2014): 5729, 10.1038/srep05729. PMC4103093 25034144

[52] L. Alderighi , P. Gans , A. Ienco , D. Peters , A. Sabatini , and A. Vacca , “Hyperquad Simulation and Speciation (HySS): A Utility Program for the Investigation of Equilibria Involving Soluble and Partially Soluble Species,” Coordination Chemistry Reviews 184 (1999): 311–318, 10.1016/S0010-8545(98)00260-4.

[53] T. Saha , P. Kumar , K. Mukhopadhyay , and S. Das , “An Investigation on *In Vitro* Bacterial Cell Permeabilization of a Zn^II^‐Curcumin Complex Along With Exploration of the Possibility of FtsZ Inhibition by *In Silico* Simulation,” Bioorganic Chemistry 181 (2026): 110302, 10.1016/j.bioorg.2026.110302. 42561563

[54] C. F. Baes and R. E. Mesmer , The Hydrolysis of Cations; Chapter 11 (Wiley, 1976), Section 11.2 (data and compilation for Cobalt(II) hydrolysis parameters and specific hydrolysis constants and stability summary for Co2+ are detailed in Table 11.2).

[55] K. J. Powell , P. L. Brown , R. H. Byrne , et al., The IUPAC Series “Chemical Speciation of Environmentally Significant Metals With Inorganic Ligands” That Covers Major Metal Ions Like Hg2+ (Part 1), Cu2+ (Part 2), Pb2+ (Part 3), Cd2+ (Part 4) and Zn2+ (Part 5) interacting with OH−, Cl−, CO32−, SO42− and PO43.

[56] M. J. Frisch , G. W. Trucks , H. B. Schlegel , et al., Gaussian, Inc., Wallingford CT (2016) GaussView 5.0. Wallingford, E.U.A.

[57] A. D. Becke , “Density‐Functional Thermochemistry. III. The Role of Exact Exchange,” Journal of Chemical Physics 98 (1993): 5648e5652, 10.1063/1.464913.

[58] C. T. Lee , W. T. Yang , and R. G. Parr , “Development of the Colle‐Salvetti Correlation‐Energy Formula Into a Functional of the Electron Density,” Physical Review B 37 (1988): 785e789, 10.1103/PhysRevB.37.785. 9944570

[59] S. Chiodo , N. Russo , and E. Sicilia , “LANL2DZ Basis Sets Recontracted in the Framework of Density Functional Theory,” Journal of Chemical Physics 125 (2006): 104107, 10.1063/1.2345197. 16999515

[60] T. Saha , S. Singha , S. Kumar , and S. Das , “Spectroscopy Driven DFT Computation for a Structure of the Monomeric Cu^2+^‐Curcumin Complex and Thermodynamics Driven Evaluation of Its Binding to DNA: Pseudo‐Binding of Curcumin to DNA,” Journal of Molecular Structure 1221 (2020): 128732, 10.1016/j.molstruc.2020.128732.

[61] J. E. B. Randles , “A Cathode Ray Polarograph. Part II.—The Current‐Voltage Curves,” Transactions of the Faraday Society 44 (1948): 327–338, 10.1039/TF9484400327.

[62] S. Doğan , A. Meşe , M. F. Baran , et al., “Anticancer, Antioxidant and Antimicrobial Activity of *Elaeagnus Angustifolia* L. leaf Extract,” Drug Design, Development and Therapy 19 (2025): 7719–7734, 10.2147/DDDT.S509705. PMC12414469 40923006

[63] H. C. Birnboim and J. J. Jevcak , “Fluorometric Method for Rapid Detection of DNA Strand Breaks in human White Blood Cells Produced by Low Doses of Radiation,” Cancer Research 41 (1981): 1989–1992. 7214357

[64] S. Das , A. Saha , and P. C. Mandal , “Radiation‐Induced Double‐Strand Modification in Calf Thymus DNA in the Presence of 1, 2‐Dihydroxy‐9, 10‐Anthraquinone and Its Cu(II) Complex,” Environmental Health Perspectives 105 (1997): 1459–1462. 10.1289/ehp.97105s61459 PMC1469930 9467063

[65] M. S. Blois , “Antioxidant Determinations by the Use of a Stable Free Radical,” Nature 181 (1958): 1199–1200, 10.1038/1811199a0.

[66] G. J. Papariello and M. A. M. Janish , “Diphenylpicrylhydrazyl as an Organic Analytical Reagent in the Spectrophotometric Analysis of Phenols,” Analytical Chemistry 38 (1966): 211–214, 10.1021/ac60234a016.

[67] J. Xie and K. M. Schaich , “Re‐Evaluation of the 2,2‐Diphenyl‐1‐Picrylhydrazyl Free Radical (DPPH) Assay for Antioxidant Activity,” Journal of Agricultural and Food Chemistry 62 (2014): 4251–4260, 10.1021/jf500180u. 24738928

[68] M. C. Foti , C. Daquino , and C. Geraci , “Electron‐Transfer Reaction of Cinnamic Acids and Their Methyl Esters With the DPPH• Radical in Alcoholic Solutions,” Journal of Organic Chemistry 69 (2004): 2309–2314, 10.1021/jo035758q. 15049623

[69] H. Wang and J. A. Joseph , “Quantifying Cellular Oxidative Stress by Dichlorofluorescein Assay Using Microplate Reader,” Free Radical Biology and Medicine 27 (1999): 612–616, 10.1016/S0891-5849(99)00107-0. 10490282

[70] O. Myhre , J. M. Andersen , H. Aarnes , and F. Fonnum , “Evaluation of the Probes 2′,7′‐Dichlorofluorescin Diacetate, Luminol, and Lucigenin as Indicators of Reactive Species Formation,” Biochemical Pharmacology 65 (2003): 1575–1582, 10.1016/S0006-2952(03)00083-2. 12754093

[71] A. Gomes , E. Fernandes , and J. L. Lima , “Fluorescence Probes Used for Detection of Reactive Oxygen Species,” Journal of Biochemical and Biophysical Methods 65 (2005): 45–80, 10.1016/j.jbbm.2005.10.003. 16297980

[72] B. Kalyanaraman , V. Darley‐Usmar , K. J. A. Davies , et al., “Measuring Reactive Oxygen and Nitrogen Species With Fluorescent Probes: Challenges and Limitations,” Free Radical Biology and Medicine 52 (2012): 1–6, 10.1016/j.freeradbiomed.2011.09.030. PMC3911769 22027063

[73] S. A. Cantalalupo , S. R. Fiedler , M. P. Shores , A. L. Rheingold , and L. H. Doerrer , “High Spin Square Planar Co^II^ and Fe^II^ Complexes and Reasons for Their Electronic Structure,” AngewandteChemie 51 (2011): 1000–1005. 10.1002/anie.201106091 22162103

[74] W. Wu , M. Huang , Q. Zeng , W. Xian , W. Liao , and J. He , “Electrical and Magnetic Properties of a Radical‐Based Co(II) Coordination Complex With C H⋯π and π⋯π Supramolecular Interactions,” Inorganic Chemistry Communications 103 (2019): 149–153, 10.1016/j.inoche.2019.03.022.

[75] F. L. Urbach , R. D. Bereman , J. A. Topich , M. Hariharan , and B. J. Kalbacher , “Stereochemistry and Electronic Structure of Low‐Spin, Square‐Planar Cobalt(II) Chelateswith Tetradentate Schiff Base Ligands,” Journal of the American Chemical Society 96 (1974): 5063–5069, 10.1021/ja00823a008.

[76] O. J. Hernández‐Ortiz , M. A. Pérez‐Guerra , and D. M. Moreno‐García , “Theoretical DFT and TDDFT Study and Experimental UV‐Vis Characterisation of Carminic Acid‐Tin Complexes; Molecular Docking Study of Metal Complexes for Potential Cancer Therapeutic Applications,” Molecular Physics 123 (2025): 2431529, 10.1080/00268976.2024.2431529.

[77] P. Nandy and S. Das , “ *In Situ* Reactivity of Electrochemically Generated Nitro Radical Anion on Ornidazole and Its Monomeric Cu(II) Complex With Nucleic Acid Bases and Calf thymus DNA,” Inorganica Chimica Acta 501 (2020): 119267, 10.1016/j.ica.2019.119267.

[78] J. Marmur and P. Doty , “Determination of the Base Composition of Deoxyribonucleic Acid From Its Thermal Denaturation Temperature,” Journal of Molecular Biology 5 (1962): 109–118, 10.1016/S0022-2836(62)80066-7. 14470099

[79] Sigma‐Aldrich, Inc ., Deoxyribonucleic Acid From Calf Thymus, Type XV, Activated, Lyophilized Powder (Sigma Chemical Company, n.d.), Product Number D4522;.

[80] O. Wangpradit , A. Rahaman , S. V. S. Mariappan , G. R. Buettner , L. W. Robertson , and G. Luthe , “Breaking the Dogma: PCB‐Derived Semiquinone Free Radicals Do Not Form Covalent Adducts With DNA, GSH, and Amino Acids,” Environmental Science and Pollution Research 23 (2016): 2138–2147, 10.1007/s11356-015-5007-4. PMC4767158 26396011

[81] M. C. Foti , C. Daquino , and C. Geraci , “Electron‐Transfer Reaction of Cinnamic Acids and Their Methyl Esters With the DPPH• Radical in Alcoholic Solutions,” Journal of Organic Chemistry 69 (2004): 2309–2314, 10.1021/jo035758q. 15049623

[82] Z. Gao , K. Huang , X. Yang , and H. Xu , “Free Radical Scavenging and Antioxidant Activities of Flavonoids Extracted From the Radix of ScutellariabaicalensisGeorgi,” Biochimica et Biophysica Acta (BBA)—General Subjects 1472 (1999): 643–650, 10.1016/S0304-4165(99)00152-X. 10564778

