# Supplementary material for: From a Cochineal Dye to the Possibility of an Anticancer Agent: Free Radical‐Mediated Activity of Carminic Acid and Its CoII Complex on Nucleobases, DNA, and PC3 Prostate Carcinoma Cells
Source: Chem Biodivers. 2026 Oct 21;23(10):e01312. doi: 10.1002/cbdv.202501312 (PMC13685477; doi:10.1002/cbdv.202501312)
Supplement: Supplementary file 1 [file CBDV-23-e01312-s001.docx]

**Supporting Information**

**From a Cochineal dye to the possibility of an anticancer agent: Free Radical-Mediated Activity of Carminic Acid and its Co^II^ complex on nucleobases, DNA and PC3 Prostate Carcinoma cells**

**^1^Tusar Kanti Mandal, ^1^Argha Dasgupta, ^1^Tanmoy Saha, ^2^Sougata Ghosh Chowdhury, ^2^Parimal Karmakar, ^1^Saurabh Das^*^**

**Department of Chemistry (Inorganic Section), Jadavpur University,**

**Kolkata-700032, India.**

**Department of Life Science and Biotechnology, Jadavpur University,**

**Kolkata-700032, India.**

**Fig. S1**

**B**

**C**

**A**

**Figure S1: (A) Plot showing a variation in absorbance at 510 nm for a change in concentration of CA at a fixed concentration of Co^II^; [Co^II^ = 50 µM]; (B) Plot showing a variation in absorbance at 510 nm for a change in concentration of Co^II^ at a fixed concentration of CA; [CA = 100 µM]; (C) a continuous variation of CA and Co^II^ at physiological pH. (~7.4); for (C), strength of stock solutions of Co^II^ and CA were 500 µM; [NaNO_3_] = 0.01 M, Temp. = 301 K.**

**Fig. S2**

**Fig. S2: Absorption spectra of CA in an aqueous solution at different pH; [CA] = 100 µM, [NaNO_3_] = 0.1 M, T = 301K.**

For CA, at low pH (3.58 to 4.92), a peak appeared at 493 nm that got shifted to higher wavelength at 521 nm at pH 5.25. This corresponds to the dissociation of a proton from the carboxyl unit in CA. Beyond this pH, in the range from 5.25 to 7.32 the peak remained more or less constant around 529 nm showing an absorbance higher than that recorded earlier. A weak shoulder was detected in the spectrum at pH 5.51 which upon further increase in pH (~7.32) developed into a peak at 529.5 nm. Intensity of this peak increased with increase in pH. Upon further increase in pH to values beyond 8.0 the absorbance corresponding to the major peak was found at 552 nm. Upon still further increase in pH to a value of 10.55, a further shift in peak occurred in the spectra; λ_max_ recorded at 568 nm. These two shifts at higher pH to higher wavelength correspond to dissociation of two phenolic-OH protons in CA suggesting formation of a new species that was followed at 568 nm for determination of pK_a_.

**Fig. S3**

**Fig. S3: Spectrophotometric titration of CA shown by a variation in absorbance at 568 nm; [CA] = 100 µM; [NaNO_3_] = 0.01 M, T = 301 K.**

**Fig. S4**

**Figure S4**: **Absorption spectra of CA in the presence of Co^II^ in an aqueous solution at different pH; [CA] = 100 µM, [NaNO_3_] = 0.1 M, Temperature = 301K.**

Co^2+^	+ 2QH_4_	Co(QH_3_)_2_	+	2H^+^	(S1)

$\text{β}\text{* = }\frac{{{[Cu(QH}_{3})}_{2}][{{H^{+}]}^{2}}}{{[Cu}^{2+}] [{{QH}_{4}]}^{2}}$	(S2)

Co^2+^	+ 2QH_3_ ^–^	Co(QH_3_)_2_	(S3)

$\text{β}\text{ = }\frac{{{[Cu(QH}_{3})}_{2}]}{{[Cu}^{2+}] [{{{QH}_{3}}^{-}]}^{2}}$	(S4)

$\text{β}\text{ = }\frac{\text{β}^{*}}{{K_{2}}^{2}}$	(S5)

**Fig. S5**

**Fig. S5: Infra-red spectra of Carminic acid (—) and Co^II^ complex of Carminic acid (—) recorded at 301 K.**

**Fig. S6**

**Figure S6**: Mass spectrum of a Co^II^ complex of Carminic acid

**Fig. S7**

**Fig. S7**: Species distribution diagrams for a Co^II^–carminic acid system as a function of pH, computed in HySS. Curves show percentage of total Co^II^ present as each species: free Co²⁺(red), the Co^II^–carminate complex (purple), CoOH⁺ (green) and Co(OH)_2_ (cyan). The dashed vertical line marks pH 7.4. **(Left)** At the concentration used in electrochemical/DNA experiments ([Co^2+^] = 4.5 µM, [CA] = 9 µM), complex accounts for ~57% of total cobalt^II^ at pH 7.4. **(Right)** At concentration used for stability-constant determination ([Co^2+^] = 50 µM, [CA] = 100 µM), complex accounts for ~90% of total cobalt^II^ at pH 7.4, illustrating the fact of a concentration dependence expected for a 1:2 metal–ligand system.

**Fig. S8**

**Fig. S8**: Frontier molecular orbitals (FMOs) of optimized geometry of carminic acid in DMSO

**Table S1: HOMO-LUMO values of Carminic Acid**

| Serial Number | Molecular Orbital | Energy (eV) |
| --- | --- | --- |
| 133 | LUMO+4 | -0.201 |
| 132 | LUMO+3 | -0.763 |
| 131 | LUMO+2 | -1.562 |
| 130 | LUMO+1 | -2.426 |
| 129 | LUMO | -3.523 |
| 128 | HOMO | -6.271 |
| 127 | HOMO-1 | -6.849 |
| 126 | HOMO-2 | -7.094 |
| 125 | HOMO-3 | -7.241 |
| 124 | HOMO-4 | -7.457 |

**Table S2: HOMO-LUMO values of Co^II^ complex for α-spin state**

| Serial Number | Molecular Orbital | Energy (eV) |
| --- | --- | --- |
| 268 | LUMO+4 | -1.801 |
| 267 | LUMO+3 | -3.228 |
| 266 | LUMO+2 | -3.395 |
| 265 | LUMO+1 | -5.035 |
| 264 | LUMO | -5.147 |
| 263 | HOMO | -6.110 |
| 262 | HOMO-1 | -6.150 |
| 261 | HOMO-2 | -6.683 |
| 260 | HOMO-3 | -6.862 |
| 259 | HOMO-4 | -6.987 |

**Table S3: HOMO-LUMO values of Co^II^ complex for β-spin state**

| Serial Number | Molecular Orbital | Energy (eV) |
| --- | --- | --- |
| 267 | LUMO+4 | -1.939 |
| 266 | LUMO+3 | -2.010 |
| 265 | LUMO+2 | -2.148 |
| 264 | LUMO+1 | -3.403 |
| 263 | LUMO | -3.643 |
| 262 | HOMO | -6.165 |
| 261 | HOMO-1 | -6.326 |
| 260 | HOMO-2 | -6.754 |
| 259 | HOMO-3 | -6.877 |
| 258 | HOMO-4 | -7.000 |

**Fig. S9**

**B**

**A**

**D**

**C**

**Figure S9**: (A) and (B) represent HPLC chromatograms recorded at 254 nm for 1 × 10^-3^ mol dm^-3^ adenine and thymine respectively after 1 × 10^-4^mol dm^-3^ CA in whose vicinity nucleobases were kept was subjected to reduction at a constant potential of -0.65 V. (C) and (D) represent HPLC chromatograms recorded at 254 nm for 1 × 10^-3^ mol dm^-3^ adenine and thymine respectively after 1 × 10^-4^ mol dm^-3^ Co(CA)_2_^2-^ in whose vicinity nucleobases were kept was subjected to reduction at a constant potential of -0.67 V. The value presented in a box for each chromatogram indicates the time for which reduction was performed at constant potential.

**Fig. S10**

**Fig. S10: Cytotoxic effect of CA (—•—) and [Co^II^(CA)_2_]^2-^ (—•—) on W I 38 normal human fetal lung fibroblast cells.**
